# Developing and integrating physician assistants/associates in UK hospital teams: a realist review of lessons from international experiences

**DOI:** 10.1186/s12916-025-04530-z

**Published:** 2025-12-29

**Authors:** Yingxi Zhao, Shobhana Nagraj, Rhys Swainston, Gerry McGivern, Tricia Tooman, Kim A. Walker, Attakrit Leckcivilize, Mike English, Geoff Wong

**Affiliations:** 1 Nuffield Department of Medicine Centre for Global Health Research, University of Oxford, S Parks Rd, Oxford, OX1 3SY UK; 2 Department of Public Health and Primary Care, University of Cambridge, Cambridge, UK; 3 East London NHS Foundation Trust, London, UK; 4 King’s Business School, King’s College London, London, UK; 5 Centre for Healthcare Education Research and Innovation, University of Aberdeen, Aberdeen, UK; 6 KEMRI-Wellcome Trust Research Programme, Nairobi, Kenya; 7 Nuffield Department of Primary Care Health Sciences, University of Oxford, Oxford, UK

**Keywords:** Additional roles, Extended roles, Task sharing, Skill mix, Team work, Workforce planning, Interprofessional teams, Legitimacy

## Abstract

**Background:**

Physician assistants/associates (PAs) were introduced into NHS secondary care facilities to help address workforce shortages in the UK. However, recent controversy and the government-commissioned Leng Review in England highlighted concerns around role clarity, supervision, and professional boundaries relating to PAs, largely due to inconsistent implementation and local variations. We examined how PA roles are developed and integrated in hospital teams across high-income countries, generating insights relevant to ongoing workforce reforms in the UK, including those recently recommended in England by the Leng Review.

**Methods:**

We conducted a realist review to explain how, why, and under what contexts PA roles are developed and integrated in secondary care. We systematically searched peer-reviewed studies from high-income settings and UK-specific grey literature (Jan 2000–March 2025). We extracted and synthesised data to develop context-mechanism-outcome configurations (CMOCs). We mapped history, regulation, and scope of practice in included countries to support contextual interpretation. We iteratively refined CMOCs to produce a final programme theory.

**Results:**

We developed 56 CMOCs from 122 sources across nine high-income settings, which were synthesised into five inter-related themes: (1) organisational drivers, such as service design, workforce shortages, and policy reforms, created opportunities for new workforce models like introducing PA roles; (2) PAs’ role and identity formation were shaped through time, supervision, and opportunities for meaningful, appropriately challenging work; (3) negotiation of professional boundaries revealed unclear or overlapping roles creating tensions, whereas well-defined, complementary roles reducing resistance; (4) role perceptions and acceptance from team members and patients depended on perceived value and relative advantages, also shaped by psychologically safe team cultures; and (5) evidence and impact were difficult to measure using standard metrics, which often overlooked PAs’ contributions to teamwork and continuity, and role variations and methodological limitations constrained generalisability.

**Conclusions:**

Our findings offer a transferrable framework for understanding workforce innovations and new roles in complex health systems. We provide practical insights for hospital managers and clinical leaders in the NHS, including those in England who are implementing the reforms recommended by the Leng Review. Realist evaluations are needed to refine our programme theory and inform effective workforce changes.

**Supplementary Information:**

The online version contains supplementary material available at 10.1186/s12916-025-04530-z.

## Background

Physician assistants (PAs) were introduced in the UK National Health Service (NHS) in the early 2000s, as part of wider efforts to expand clinical capacity and address workforce shortages [1–4]. The role was originally modelled on the US physician assistant profession, which has a longer history and more established regulation. In the UK and US, the title was changed from ‘assistant’ to ‘associate’ in 2013 and 2021 respectively [4, 5]. The role is UK-wide, with a national curriculum aligned to the General Medical Council’s generic and shared outcomes for PAs and a national exam [4]. Throughout the UK, there was an intention to increase the number of PAs but the implementation of this policy varied across the four nations. Over time, PAs’ role in hospital settings has grown but not without controversy [6]. Concerns over role clarity, supervision, patient safety, and impact on medical training triggered strong opposition to PAs from certain sectors of the medical profession and sparked wider public and political debate [7–10].

In response, the UK government commissioned the independent Leng Review (2025) to assess the PA and anaesthesia associate roles in England. The review concluded that while the PA role should be retained, it requires substantial reform [6]. The government has since welcomed and accepted all 18 recommendations the Leng Review made [11], including renaming the role back to ‘physician assistant’, strengthening regulation and supervision, and requiring all new PAs to undertake at least two years in secondary care before working in primary or mental health care settings. These policy changes apply specifically to England, although all four nations are now considering this.

Now the national policy direction relating to PAs has been clarified, it is time to consider how these reforms can be implemented in practice. The Leng Review highlighted substantial variations and inconsistencies in the way PAs are deployed across hospital trusts, often in the absence of defined role expectations, supervision structures, and integration strategies [6]. These inconsistencies contributed to confusion and resistance, yet also offer learning opportunities for hospital managers and clinical leaders to implement the recommended reforms more effectively.

We explain how, why, and under what contexts PA roles are successfully developed and integrated into hospital-based teams. Drawing upon evidence from high-income settings, we explore contextual factors, mechanisms, and outcomes that shape the development of the PA role, and its implementation in secondary care. Realist review methodology is well suited to this, as it focuses on understanding how different contexts trigger specific mechanisms that influence outcomes [12, 13], which aligns with the complexity and variability of PA implementation across healthcare settings. This approach has been successfully applied in previous workforce topics, including paramedics [14], link workers [15], workforce wellbeing [16], and workforce behaviour [17], demonstrating its value in unpacking the dynamic, relational, and context-dependent nature of workforce innovation and changes.

We deliberately focus on secondary care settings, where the majority of PAs in the UK are employed, and in which the impact of the new two-year early career requirement will be most significant [6]. Our emphasis is on the organisational and workforce dimensions of PA role development and integration, how hospital teams make sense of, support, and embed the role, rather than on the clinical effectiveness or safety, which remains poorly evidenced and was recognised as difficult to assess in the Leng Review [6]. By synthesising the evidence through an explanatory lens, we offer practical insights for hospital managers and clinical leaders tasked with embedding PAs into clinical teams in line with current policy reforms. Our synthesis of evidence also offers transferrable lessons for other countries and settings, where similar new roles are being introduced and scaled.

## Methods

Realist review is a theory-driven interpretative approach to synthesising existing data. Grounded in realist philosophy, realist review aims to explain how and why outcomes occur by examining how specific contexts (settings and conditions) trigger mechanisms (latent or often invisible property of a person, object, or institution) [12, 13]. We developed and tested (confirmed, refuted, or refined) context-mechanism-outcome configurations (CMOCs) to generate a transferrable programme theory, which is essentially an abstract description or diagram that sets out what a programme comprises and how it is expected to work [18]. Programme theories are useful because they make explicit the assumptions about why an intervention works, for whom, and under what circumstances. Our review builds on our earlier scoping review that focused on advanced practice providers, including PAs and nurse practitioners [19], by offering explanatory insights into what works, for whom, in what circumstances, and why.

We followed the Realist and Meta-Review Evidence Synthesis: Evolving Standards (RAMESES) standards [18, 20]. Our review was undertaken between March 2024 and June 2025, notably prior to the publication of the Leng Review, and the protocol was registered on PROSPERO (CRD42024528814). The review team comprised health systems researchers, realist researchers, and clinicians working on a broader study of PAs in UK NHS hospitals. While no team member is a practising PA, the review was enriched by feedback and advice on the emerging findings from a broader collaborator group, including practising PAs in secondary care, educators, supervisors, and managers of PAs in higher education and clinical settings, policy stakeholders (e.g. regulators), and a dedicated patient and public involvement (PPI) group. These outsider-insider views [21], along with concurrent empirical data collection and analysis, contributed to the refinement and validation of our findings.

### Step 1: locate existing theories

We drew on our scoping review [19] to develop the initial programme theory and identify relevant substantive theory related to PA role development and integration in secondary care. This initial programme theory was refined through internal discussions and external feedback from collaborators and the PPI group.

### Step 2: search for evidence

Seventy-five peer-reviewed articles were identified through our earlier scoping review [19], which systematically searched five databases from January 2000 to April 2023 (Ovid MEDLINE, Ovid Embase, Ovid Global Health, Ovid PsycINFO, and EBSCOhost CINAHL). We only included studies that explicitly focused on PAs.

An updated search was conducted in March 2025 using the same search strategy (see Additional file 1) to capture more recent publications. To supplement this, we also conducted a UK-focused grey literature search, recognising the salience of this topic and the role of grey sources in explaining how interventions work in practice [18]. We reviewed stakeholder reports and statements from 22 organisations (see Additional file 2), including Department of Health and Social Care, NHS England, General Medical Council, British Medical Association, different medical royal colleges, and United Medical Associate Professionals. Only grey literature with empirical content was included. YZ conducted all update and grey literature searches.

### Step 3: select studies

Our selection was based on relevance to the programme theory and rigour. We focused primarily on empirical studies that examined the development and integration of PA roles in secondary or hospital care in high-income countries. Opinion pieces and commentaries were excluded unless they offered insights into how PA roles were developed in specific hospital settings. For grey literature, we included only empirically grounded content, such as member consultation data or systematically collected qualitative data (e.g. British Medical Association members portal submissions [22]). YZ screened titles and abstracts using Rayyan.ai software and full texts using Microsoft Excel.

### Step 4: extract and organise data

All included documents were imported into NVivo for data extraction. Key characteristics of the sources such as country, study design, and participants were extracted into a Microsoft Excel spreadsheet (see Additional file 3) [5, 22–142]. We coded the data both deductively and inductively. Deductive coding was guided by abstract categories derived from our initial programme theory, while inductive coding allowed specific sub-categories and new categories to emerge from the data. Coding was conducted by YZ and emerging causal explanations (in the form of a narrative and/or CMOCs) were regularly shared with GW, who provided further insights into the veracity (drawing on the included data) and phrasings of these explanations.

We first coded the 75 studies drawn from the earlier scoping review, and then coded the updated and grey literature sources. To support contextual interpretation of the data, we also conducted background searches for each included country or territory (and relevant subnational level) to map the history, regulation, and scope of practice of PA roles (see Additional file 4).

### Step 5: synthesise the evidence according to a realist logic of analysis

We synthesised data using realist logic of analysis, by comparing and configuring the coded data within and across studies. We developed CMOCs iteratively by identifying outcome patterns, contextual factors, and using retroductive reasoning to infer underlying mechanisms [143]. We used further substantive theories to support or refine interpretations.

Over the course of synthesis, we clarified that our realist review’s central aim was to understand if and how PA roles could be effectively developed and integrated within UK NHS hospitals. Accordingly, we considered national-level factors (e.g. regulation, policy guidance) as contextual conditions rather than outcomes of interest. For example, we did not examine whether a national scope of practice should be introduced, but rather explored how tightly or loosely defined scopes shaped local role implementation and perceptions.

YZ and GW collaboratively reviewed each CMOC, as well as three data excerpts, receiving feedback from the broader research team, and several other external realist health systems researchers. We developed the final programme theory by integrating CMOCs into a coherent narrative, which was shared with collaborators and presented at two UK and international health services research conferences, and then underwent further refinement.

## Results

### Overview of sources

**Fig. 1:**
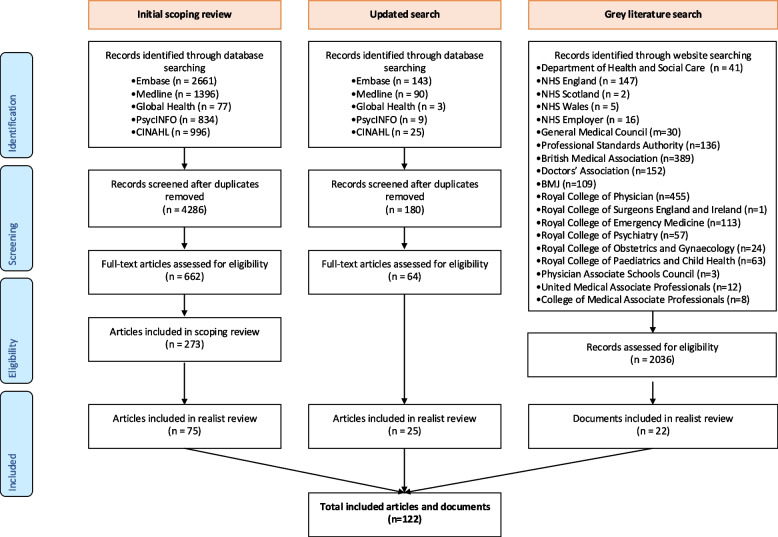
Realist review PRISMA flow diagram providing summary of searching and selection process

From the initial scoping review, updated and grey literature search, 122 sources were identified and included in our analysis. The PRISMA diagram is shown in Fig. 1. These sources span 2000–2025 and originate from the US (63 studies across 20 states, territories, and national-level sources), UK (18 studies and 22 grey literature sources), Canada (seven studies from three provinces and national-level), Netherlands (five studies), Australia (two studies from two states), Ireland (two studies), and Israel, Taiwan, and South Korea (one study each). The included sources represent a range of clinical settings. Eighteen focused on inpatient care, three on outpatient, 21 on emergency departments, one on ambulatory care, and the rest examined mixed settings. In terms of study design, 69 were quantitative, 37 were qualitative, and 16 used mixed methods.

Drawing on this evidence base, we developed 56 CMOCs, which we grouped into five overarching themes: organisational drivers; role and identity formation; boundary work; role perception and acceptance; and evidence and impact. Although these five themes are presented below in a linear manner for clarity, in practice there is substantial overlap and interdependence across them. Role and identity formation, boundary work, and role perception and acceptance often occur simultaneously, and feedback loops between the rest are common.

We summarise the 56 CMOCs from the analysis below. Additional file 5 provides further detail of each CMOC, supporting data excerpts, and all the sources that support each CMOC.

### Organisational drivers

The decision to develop PA roles is shaped by how hospital and clinical department leaders perceive workforce needs and priorities. When dissatisfied with the status quo or pressured to resolve the service delivery challenges, organisations are more likely to explore new workforce models, including new roles like PAs (CMOC A1). In contrast, when the status quo is seen as sufficient, or other competing staffing priorities dominate (such as expanding advancing nursing practice), the PA role is less likely to gain support (CMOC A2, A6). National political endorsement and clear policy signals can strengthen organisational confidence and create legitimacy for the role (CMOC A3). Additionally, organisations and clinical teams with prior positive exposure to PA roles, or opportunities to observe the roles in action, are more likely to consider them (CMOC A4). Conversely, organised professional resistance, particularly from influential medical bodies, can discourage confidence in and adoption of PA roles (CMOC A5). The absence of long-term workforce planning for PAs further impedes this, as there may be no clear direction, commitment, or resource to support new roles (CMOC A7).

Financial practicality also matters. Earmarked and accessible funding sources make it easier to commit to the role (CMOC A8), while financial ambiguity makes it unclear whether to prioritise and sustain the role (CMOC A9). The availability of qualified PAs locally, especially those with relevant specialised clinical expertise meeting local service needs, also influences whether organisations can recruit and embed the PA role effectively (CMOC A10). Leaders must weigh all these factors and construct a compelling clinical and financial case for investment in developing PA roles within their organisations (CMOC A11). Even with these conditions in place, the development and implementation still requires dedicated individuals and champions to devote time and energy to drive advocacy, engagement, and sustained momentum (CMOC A12).

These dynamics are underpinned by whether the *institutional legitimacy* of new roles can be strategically managed and socially conferred [144, 145]. The development of the PA role not only involves responding to external policy signals, but also navigating internal structures, such as actively interpreting service pressures, resource availability, and interprofessional dynamics. Our findings also highlighted the importance of *organisational alignment* [146] with new roles. PAs are more likely to develop when aligned vertically (with national policy and leadership objectives) and horizontally (with structures, workflows, and priorities of professional groups, departments, and clinical teams). When new roles are perceived as imposed by external authorities or senior organisational leaders, without local consultation, or as misaligned with existing priorities or structures, they are more likely to face resistance from the local workforce and fail to embed.

### Role and identity formation

This theme explores how PAs come to understand, shape and enable their roles, together with their teams, and the way PAs navigate uncertainty, build competence, and form professional identities within clinical teams. When departments and clinical teams have the authority to determine staffing and roles, they can adapt PA roles to suit local needs and service gaps, such as taking on specialised tasks or contributing to team-based working (CMOC B1). However, without clear expectations, consistent communication, and defined lines of accountability, role development for PAs becomes difficult and emotionally challenging (CMOC B2-B4). These expectations need to be clearly articulated by department leaders but also communicated in ways that are understood and reinforced by all team members. Frequent staff turnover can undermine this process, as a lack of team continuity leads to loss of shared understanding about PA roles (CMOC B5).

Time and supportive supervision also play a part in role and identity formation. Supervisors who invest time in guiding PAs through workplace hierarchies and norms help create a pathway for development and recognition (CMOC B6), whereas the absence of regular feedback and appraisal can leave PAs uncertain about their role and identity (CMOC B7). When given sufficient time to adjust and demonstrate their abilities, PAs often gain their clinical colleagues’ trust, as clinical team members are able to observe and form clearer judgements about their capabilities in practice (CMOC B8-B10).

PAs’ capacity to shape and grow into their roles is also influenced by prior experience and new opportunities. PAs with previous clinical or healthcare experience (e.g. having previously worked as a pharmacist or paramedic) tend to adjust more quickly and navigate team relationships more effectively (CMOC B11). Opportunities for skill-building and engaging in challenging work contribute to a sense of accomplishment and identity affirmation (CMOC B12), whereas being restricted to undesirable tasks (such as those that are perceived as low-skill, repetitive, offer little opportunity for learning) may leave PAs feeling undervalued and dissatisfied (CMOC B13).

These findings align with *role development theory*, which explains how occupational roles are continually constructed through social interaction and behavioural negotiations within teams and organisations [147] like when PAs are given time and space to grow into responsibilities and adapt to local service needs. In parallel, *identity theory* helps explain how individuals form and maintain a sense of who they are within specific roles, and the way they seek validation of this identity from others [148]. PAs often enter their role with internalised expectations, and external feedback can either reinforce or undermine this sense of self. When external validation undermines their sense of self (e.g. when they are confined to administrative functions or ‘scut work’ [149]), identity disruption can occur, for example leaving PAs feeling marginalised or disconnected from their professional role.

### Boundary work

This theme focuses on how introducing new roles, like PAs, can disrupt established professional boundaries. In settings where PAs are perceived as lacking experience, competence, or understanding of the healthcare system, organisations and individuals may question the value of investing in them (CMOC C1). These doubts may be reinforced when their roles are narrowly defined or restricted by regulations, governance, or internal protocols, leading to perception of their limited benefit (CMOC C2).

Conversely, when the policy and regulatory environment is more flexible, department and clinical team leaders have discretion to shape the responsibilities around service needs (CMOC C3). In settings facing acute staffing challenges (e.g. rural areas, high patient demand, or emergencies), PAs often take on broader scopes of practice out of necessity (CMOC C3). However, this flexibility can generate ambiguity. Unclear or overlapping responsibilities between PAs and other health professional groups can blur role boundaries and create confusion about the purpose of the PA roles (CMOC C5). These tensions heighten when overlaps are seen as encroaching on the jurisdiction of other existing health professions, particularly when these professionals, such as resident doctors and advanced practice nurses (a category that is known to encompass multiple overlapping titles [150]), are also seeking to consolidate or expand their own scope of practice (CMOC C6). Conflicting and unrealistic expectations from clinical team members further complicates how PAs are perceived and integrated into clinical teams (CMOC C7).

Access to shared organisational resources, such as supervision, training opportunities, and funding, can be another point of contention in professional boundary negotiations. When PAs are seen as competing for scarce resources, or having organisational advantage over other clinical team members, such as higher salary or more favourable working hours, this can create resistance to their introduction from other health professions (CMOC C8, C9). By contrast, when their role is perceived as non-threatening and complementary to existing professional ways of working, such as extending previously unachievable clinical service functions, or taking on less desirable or low-priority clinical tasks that other professionals are unable or unwilling to fill, PAs are more likely to gain recognition and acceptance (CMOC C10, C11).

Abbott’s theory relating to *jurisdiction within the system of professions* [151] and the theory of *negotiated order* [152] reflect these findings, illustrating how professions claim control over specific domains of work, and the way tensions can arise when those claims are challenged. Whether the introduction of PA roles is accepted or resisted by other health professionals depends on how these jurisdictional boundaries are settled, whether through subordination, cliental differentiation (e.g. managing lower-acuity patients or only in particular settings like inpatient wards), or intellectual differentiation (e.g. contributing specialist knowledge in team continuity and system navigation). Crucially, these boundaries are fluid and continuously reshaped through everyday interactions and negotiations in the workplace. This is important in clinical environments, where other professional roles, such as resident doctors and advanced practitioners, are also evolving. In such settings, the integration of PAs is influenced as much by informal, day-to-day negotiations, as by formal job descriptions.

### Role perception and acceptance

This section examines how PAs come to be recognised as legitimate members of the clinical team and as legitimate by the public. When clinical team members have no or limited exposure to PA roles, confusion about their role can lead to reluctance and resistance to their introduction (CMOC D1). Acceptance is more likely when powerful internal stakeholders help define PAs’ responsibilities (CMOC D2) and when the PA role is institutionalised through policies, procedures, and leadership structures (CMOC D3). Individual PA’s credibility also contributes to this. PAs with prior health professional experiences (CMOC D4), or those who clearly, approachably, and professionally communicate their role (CMOC D5), are more likely to be accepted. By contrast, strained relationships can undermine PAs’ acceptance by other professionals, and their own sense of professional satisfaction and belonging (CMOC D6). Supportive and psychologically safe team cultures promote openness, encouraging PAs and colleagues to raise concerns and address challenges, leading to easier acceptance of PAs (CMOC D7). Once established, PAs may be trusted, but this can unintentionally limit learning opportunities as PAs are relied upon over newer team members (CMOC D8).

Perceptions of legitimacy can also extend to patients and the public. We drew most of these insights directly from patients reports, though some reflect professionals’ perceptions of what patients may think (see Additional file 4). When PA roles are poorly differentiated from other clinicians, public confusion can arise about their role and scope of practice (CMOC D9). Concerns about PAs’ competence may reduce patients’ confidence in their ability to provide safe and effective care (CMOC D10). Trust in the broader healthcare team can offset the uncertainty of the role (CMOC D11). Where patients see clear advantages, such as quicker access or more time during consultation, they may prefer to see a PA (CMOC D12).

These dynamics reflect principles of *relational coordination*, especially how shared goals, mutual respect, and open communication support the integration of new roles [153, 154]. The successful acceptance of PAs often hinges on the strengths of interpersonal relationships, their ability to resolve tensions openly and collaboratively, involving the creation of psychologically safe environments, where team members can raise concerns and clarify misunderstandings. They also align with *diffusion of innovation theory* [155], which suggests that one’s perception of relative advantage (e.g. improved continuity or access), compatibility with existing ways of working, and complexity may influence the acceptance of new roles like PAs.

### Evidence and impact

Sustained integration also depends on how the value and contribution of the PA role is evidenced, interpreted, and used to inform ongoing organisational support. When hospitals and departments observe that PAs save resources, improve efficiency, or contribute to other positive outcomes (such as continuity and staff experience), they are more likely to sustain the PA role (CMOC E1, E2). However, isolating and quantifying PAs’ individual contributions is often challenging. PAs usually work within larger multidisciplinary teams, and service performance and patient outcomes are typically measured at the team or service level, not the individual or professional level (CMOC E3). PAs’ contributions, such as improving continuity and interprofessional collaboration, are also difficult to capture. These outcomes are often described subjectively and qualitatively and are hard to measure using traditional quantitative indicators (CMOC E4).

Further difficulties arise due to the variation in how PAs are deployed across settings (CMOC E5) and the use of mixed and sometimes conflicting outcomes metrics (e.g. service delivery, efficiency, patient satisfaction, and health outcomes) complicates interpretation of PAs’ overall impact (CMOC E6). For example, results within and across studies could be conflicting. Some suggest PAs improve costs or service efficiency, while others report increased length of stay or no clear benefit. These inconsistencies create confusion about PAs’ impact. Furthermore, these challenges are compounded by the inherent methodological challenges of several workforce intervention studies that are heavily confounded by local contexts and difficult to randomise or control (CMOC E7). In many settings, the absence of robust systems to track PA activities and outcomes limits organisations’ ability to generate transparent and meaningful evidence (E8).

*Normalisation Process Theory (NPT)* [156] speaks to PA role development and integration across the five themes. The development and integration of PAs depends not only on whether their role makes sense to other health care staff (coherence), and is actively supported (cognitive participation and collective action), but also whether the work they do is visible, measurable, and appraised as valuable (reflexive monitoring). When organisations struggle to monitor or interpret PAs’ contributions, the role may fail to be normalised and sustained, despite otherwise strong engagement.

### Final programme theory

**Fig. 2:**
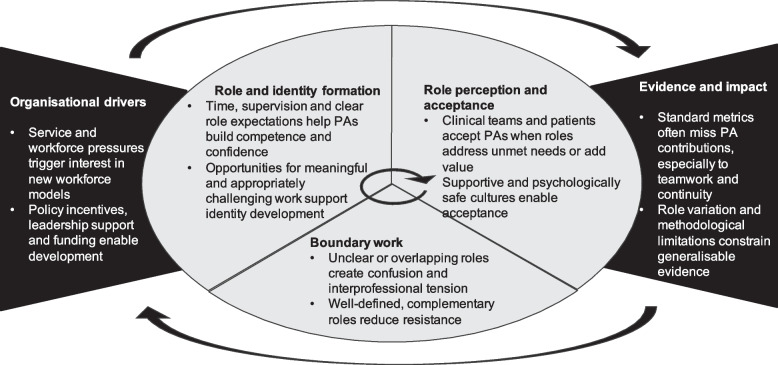
Final programme theory

Figure 2 and Table 1 present the final programme theory that explains how new roles like PAs are developed and integrated within clinical teams. The programme theory is supported by 56 CMOCs and draws on nine substantive theories to explain the underlying mechanism and contextual interactions involved.

**Table 1:** Narrative of the final programme theory

Hospitals and clinical teams develop PA roles when organisational pressures such as workforce shortages and enabling conditions (e.g. policy incentives, local leadership support, or access to funding) create a window of opportunity. However, the extent to which PA roles are developed and integrated depends on how clearly their local responsibilities are defined, whether clinical team members understand and support their integration, and whether PAs are given sufficient time and support to build their competence and confidence within the local health system. Professional boundaries and perceptions of legitimacy shape how clinical teams and patients respond to PAs. When PA roles are seen as overlapping with or competing against established professions, other health professionals may resist their implementation. However, when PAs are perceived as complementing team functions or addressing service gaps that other roles are unable or unwilling to fill, PAs are more likely to be accepted and valued. Local flexibility in defining PA roles can help tailor them to differing circumstances, but can also introduce variation that complicates wider understanding and policy and regulatory level standardisation. These variations - combined with fragmented data systems and methodological limitations - constrain the evidence base on PA impact, making it harder for decision-makers to draw generalisable conclusions informing strategic workforce planning.	

## Discussion

This review builds on and moves beyond our earlier scoping review [19] by offering further explanatory insights into how and why PAs are developed and integrated into hospital teams. We identified 56 CMOCs, grouped under five inter-related themes: *organisational drivers*; *role and identity formation*; *boundary work*; *role perception and acceptance*; and *evidence and impact*. Together, these categories provide a transferrable framework for explaining how organisational and professional conditions shape PA role development and integration in high-income secondary care settings.

Our findings complement, provide additional explanations, and operationalise key recommendations from the Leng Review [6], particularly those related to setting a clear vision, fostering local leadership and engagement, and supporting implementation through structured change management. In practice, successful PA integration is unlikely to be the product of national policy alone, but depends on how organisations interpret and embed the PA role within existing clinical and professional structures, cultures, and pathways. This reflects Lipsky’s Street-Level Bureaucracy theory [157], which highlights how frontline leaders and managers exercise discretion in implementing policy, inevitably producing local variation in how the PA role is deployed. Alongside this policy implementation variation, roles and professional boundaries are by nature fluid, actively and continually negotiated within clinical teams [2]. Confidence in new roles also tends to grow with time, direct exposure and experience, as team members develop more informed judgement about their value and contribution [158]. This echoes broader implementation theories such as Normalisation Process Theory [156], where coherence (making sense of a new role), legitimation (gaining acceptance), and collective action (embedding within teams) all influence whether a role becomes integrated and sustained.

Based on these findings, we make the following recommendations for hospital managers and clinical leaders when integrating PAs into hospital settings:

1. *Be realistic about time and support needed for change*: Introducing and integrating new roles like PAs will not be a quick fix. It requires dedicated time, resources, and leadership to redesign governance structures, supervision arrangements, patient pathways, and triage protocols [159]. It also requires strategic thinking about service delivery arrangement, since frequent changes to services models and team structures can disrupt role development. These efforts need to be supported by structured change management; without it, implementation of new PA roles risks generating confusion, resistance, and unintended consequences for staff and patients. Leaders should ensure staff have a shared understanding of the role, legitimise it through clear policies and visible leadership support, and embed it into routine practice through supervision, governance, and established pathways.
2. *Clarify role vision and expectations*: Leaders and managers should develop a clear rationale for introducing new roles such as PAs, grounded in workforce planning and understanding of available alternatives. Role expectations must be communicated consistently to clinical team members. This is particularly important in settings where there is constant turnover of clinical staff on medical training rotations, and where other evolving roles, like advanced clinical practitioners, are present. Both these groups are also navigating role development and boundaries in health systems already marked by workforce strain and dissatisfaction [7]. Clear communication with patients is equally important to prevent confusion or distrust, particularly against a background of heightened media controversy.
3. *Support PAs’ development*: PAs need both time to develop their role and competence, and opportunities for meaningful, appropriately challenging work and professional growth. Being stuck in routine, repetitive tasks [149] can undermine identity formation, affect wellbeing, and lead to poor retention. Unlike many other professional groups, PAs currently have no clearly defined career pathway, making structured development support particularly important. The Leng Review recommended exploring advanced PA roles and career pathways [6]. However, this will only be successful if leaders and managers also consider and balance the development needs of other professional roles, and support them by sustained investment in supervision, mentoring, and structured development opportunities across the multidisciplinary team.
4. *Monitor safety and effectiveness*: While the Leng Review emphasised national monitoring of patient safety in relation to PAs [6], our review highlights the importance of better understanding PAs’ broader impact. Monitoring and feeding back how PAs contribute to patient care, service delivery, and team functioning is essential for building their legitimacy and sustaining support. Yet, such monitoring is methodologically complex, as highlighted in our final group of CMOCs. Workforce interventions are complex and difficult to implement in tightly controlled conditions, and thereby, challenging to evaluate through traditional randomised study designs. Here, managers and leaders might draw upon principles of process evaluation of complex interventions when trying to evaluate PA impact [160]. Hospital systems should also strengthen routine data collection to document and assess PA contributions to health service delivery, and impact on patient experience and outcomes.

Our findings have important implications for other new or evolving roles, such as surgical care practitioners (registered healthcare professionals such as theatre nurses or operating department practitioners with extended scope) [161], nursing associates, and anaesthesia associates (to be renamed as physician assistants in anaesthesia) in hospital teams. With multiple new roles being introduced concurrently, organisations need to consider their capacity to manage and embed new roles effectively, especially managing role expectations and areas of role overlap or distinction. Some of these considerations are also relevant in primary care, for example schemes such as the Additional Roles Reimbursement Scheme in England have been used to rapidly introduce new staff groups [162].

By using a realist approach, we synthesised diverse and contextually variable evidence into a coherent explanatory framework. However, this study has limitations. First, while we limited our studies to high-income settings, a significant proportion of studies were from the US. As the Leng Review noted, US experiences are not directly transferrable to the UK, due to different funding and subnational regulatory structures [6]. However, an advantage of the realist approach is a focus on mechanisms allowing us to extract relevant explanatory insights.

Second, the evidence base remains limited regarding the detailed accounts of implementation experiences. While we included grey literature from the UK, many NHS hospitals may be reluctant to publish negative or reflective accounts of their experiences, especially if implementation was difficult, contested, or unsuccessful. Conversely, some recent grey literature and reports may overemphasise challenges or opposition, particularly in light of heightened public and professional debates. These dynamics present difficulty in capturing a balanced, accurate, and comprehensive picture of how the PA role is being embedded in practice.

Third, evidence of how PA roles vary across specialties and hospital settings remains limited. In some settings, PAs are taking on highly specialised tasks, while others contribute generalised skills linked to continuity or team-based working. These differences probably reflect local service needs but clearer understanding is needed of how and why such variation occurs. Future research should include the use of evaluation methods or approaches that are able to capture the complexities of the PA implementation within NHS trusts (e.g. realist evaluation).

## Conclusions

We synthesised global evidence to explore the development and integration of PA’s roles within hospital teams, focusing on the underlying reasons and processes. We identified 56 CMOCs across five inter-related themes (*organisational drivers*; *role and identity formation*; *boundary work*; *role perception and acceptance*; and *evidence and impact*) highlighting the importance of role clarity, professional boundaries, and organisational support. Our findings offer a transferrable framework for understanding workforce innovations in complex health systems, and practical recommendations for hospital managers and clinical leaders to implement policy reforms. Future research examining the implementation processes relating to new health professional roles, using approaches that capture the complexity involved, particularly in NHS hospital settings, is needed to support effective role integration and inform sustainable workforce development strategies.

## Supplementary Information

Additional file 1. Example of search strategy.

Additional file 2. Grey literature search.

Additional file 3. Characteristics of included sources.

Additional file 4. Background information of the nine countries and territories including states and provinces that govern PA practices separately.

Additional file 5. List of CMOC, representative quotes, and supporting data.

## Data Availability

All data relevant to the study are included in the article or uploaded as additional files.

## Abbreviations

CMOC: Context-mechanism-outcome configuration
NHS: National Health Service
PA: Physician assistants
RAMESES: Realist and Meta-Review Evidence Synthesis: Evolving Standards
