# Supplementary material for: Developing and integrating physician assistants/associates in UK hospital teams: a realist review of lessons from international experiences
Source: BMC Med. 2025 Dec 29;23:707. doi: 10.1186/s12916-025-04530-z (PMC12751915; doi:10.1186/s12916-025-04530-z)
Supplement: Supplementary file 1 — Additional file 1. Example of search strategy. [file 12916_2025_4530_MOESM1_ESM.docx]

**Additional File 1. Example of search strategy**

Database(s): Embase 1974 to present

| **#** | **Searches** | **Results** |
| --- | --- | --- |
| 1 | career/ | 36683 |
| 2 | exp workforce/ | 71251 |
| 3 | (career or workforce or employment or absenteeism or recruit* or retention or turnover* or leave or integrating or integration).ti,ab. | 1879308 |
| 4 | 1 or 2 or 3 | 1944568 |
| 5 | ("non-physician clinician*" or "nonphysician clinician" or "mid-level provider*").ti,ab. | 716 |
| 6 | ("physician assistant*" or "physician associate*" or "assistant medical officer*" or "clinical assistant*" or "anaesthesia assistant*" or "anesthesia assistant*" or "anaesthesia associate*" or "anesthesia associate*" or "surgical care practitioner*").ti,ab. | 8508 |
| 7 | 5 or 6 | 9151 |
| 8 | exp secondary health care/ | 14200 |
| 9 | exp hospital/ | 1551210 |
| 10 | (hospital* or "acute care" or "secondary care" or "tertiary care" or outpatient or "clinical team*" or "emergency department*" or NHS or UK).ti,ab. | 3464020 |
| 11 | 8 or 9 or 10 | 4017702 |
| 12 | 4 and 7 and 11 | 712 |
| 13 | limit 12 to yr="2023 -Current" | 143 |
