# Supplementary material for: Developing and integrating physician assistants/associates in UK hospital teams: a realist review of lessons from international experiences
Source: BMC Med. 2025 Dec 29;23:707. doi: 10.1186/s12916-025-04530-z (PMC12751915; doi:10.1186/s12916-025-04530-z)
Supplement: Supplementary file 2 — Additional file 2. Grey literature search. [file 12916_2025_4530_MOESM2_ESM.docx]

**Additional File 2. Grey literature search**

| Organisation | Search date | Search hit for physician associate | Comment | Included |
| --- | --- | --- | --- | --- |
| DHSC | April 27, 2025 | 41 |  | 2 |
| House of Commons Health and Social Care Committee | April 27, 2025 | 0 |  | 0 |
| NHS England | April 27, 2025 | 147 |  | 0 |
| NHS Scotland | April 27, 2025 | 2 |  | 0 |
| NHS Wales | April 27, 2025 | 5 |  | 0 |
| HSC Northern Ireland | April 27, 2025 | 0 |  | 0 |
| NHS Employer | April 27, 2025 | 16 |  | 0 |
| GMC | April 27, 2025 | 300 |  | 4 |
| PSA | April 27, 2025 | 136 |  | 0 |
| BMA | April 27, 2025 | 389 | End at page 40 | 6 |
| Doctors' Association | April 27, 2025 | 152 |  | 1 |
| Academy of medical royal colleagues | April 27, 2025 | 3 |  | 0 |
| BMJ | April 27, 2025 | 109 |  | 2 |
| RCP | April 27, 2025 | 455 |  | 1 |
| Royal college of surgeons England and Ireland | April 27, 2025 | 1 |  | 0 |
| RCEM | April 27, 2025 | 113 |  | 0 |
| Royal college of psychiatry | April 27, 2025 | 57 |  | 0 |
| RCOG | April 27, 2025 | 24 |  | 0 |
| RCPCH | April 27, 2025 | 63 |  | 6 |
| Physician Associate Schools Council | April 27, 2025 | 3 |  | 0 |
| UMAPs | April 27, 2025 | 12 |  | 1 |
| CMAPs | April 27, 2025 | 8 |  | 0 |
