# Supplementary material for: Developing and integrating physician assistants/associates in UK hospital teams: a realist review of lessons from international experiences
Source: BMC Med. 2025 Dec 29;23:707. doi: 10.1186/s12916-025-04530-z (PMC12751915; doi:10.1186/s12916-025-04530-z)
Supplement: Supplementary file 3 — Additional file 3. Characteristics of included sources. [file 12916_2025_4530_MOESM3_ESM.docx]

**Additional File 3. Characteristics of included sources**

| **ID** | **Year** | **Country** | **Cadre** | **Setting** | **Study design** | **Study design detail** | **Participants** | **title** |
| --- | --- | --- | --- | --- | --- | --- | --- | --- |
| [23] | 2019 | UK - England | PA | Inpatient | Mixed methods | Survey, focus group and interview | Consultants, junior doctors, senior nurses, PAs | 'What can you do then?' Integrating new roles into healthcare teams: Regional experience with physician associates |
| [24] | 2010 | US - Nationwide | Mixed | Emergency | Quantitative | Survey | Clinical director | A national survey: acceptance of physician assistants and nurse practitioners in trauma centers |
| [25] | 2022 | Ireland | PA | Mixed | Quantitative | Questionnaire survey | Hospital management | A survey exploring factors affecting employment of physician associates in Ireland |
| [26] | 2021 | US - Nationwide | Mixed | Mixed | Quantitative | Secondary data analysis | N/A | Advanced Practice Providers Utilization Trends in Otolaryngology From 2012 to 2017 in the Medicare Population |
| [27] | 2014 | US - Nationwide | Mixed | Mixed | Quantitative | Survey | Hospital management | Advanced practice registered nurses and physician assistants in sleep centers and clinics: a survey of current roles and educational background |
| [28] | 2019 | US - Nationwide | Mixed | Mixed | Quantitative | Survey | Paediatric surgeon | Advanced providers in pediatric surgery: Evaluation of role and perceived impact |
| [29] | 2011 | US - Florida | Mixed | Inpatient | Qualitative | Reflection | N/A | Advanced registered nurse practitioners and physician assistants in the practice of pediatric neurosurgery: a clinical report |
| [30] | 2005 | Taiwan | PA | Mixed | Quantitative | Survey | PA | An analysis of job satisfaction among physician assistants in Taiwan |
| [31] | 2012 | UK - England | PA | Mixed | Qualitative | Reflection | N/A | Cardiovascular innovations: role, impact and first-year experience of a physician assistant |
| [32] | 2016 | Netherlands | PA | Inpatient | Qualitative | Interview | Doctor, resident, nurse, PA | Determinants of the sustained employment of physician assistants in hospitals: A qualitative study |
| [33] | 2021 | US - Tennessee | Mixed | Mixed | Mixed methods | Survey and focus groups | NP, PA | Development and testing of an advanced practice clinical advancement program within an academic medical center |
| [34] | 2010 | Canada - Manitoba | PA | Inpatient | Quantitative | Secondary data analysis and survey | Doctors, nurses and patients | Experience with physician assistants in a Canadian arthroplasty program |
| [35] | 2019 | US - Texas | PA | Mixed | Qualitative | Interview | PA and doctor | Exploring the effect of PAs on physician trainee learning: An interview study |
| [36] | 2023 | US - Florida | Mixed | Inpatient | Quantitative | Survey | PA and APRN | Factors Affecting Turnover of Advanced Practice Providers: A University Teaching Hospital Review |
| [37] | 2005 | US - Pennsylvania | Mixed | Mixed | Quantitative | Survey | Surgical resident, NP and PA | How do surgical residents and non-physician practitioners play together in the sandbox? |
| [38] | 2015 | Canada - Ontario | PA | Mixed | Quantitative | Case-control | N/A | Improving health care efficiency through the integration of a physician assistant into an infectious diseases consult service at a large urban community hospital |
| [39] | 2006 | US - Virginia | Mixed | Mixed | Qualitative | Reflection | N/A | Integrating midlevel practitioners into a teaching service |
| [40] | 2020 | UK - England | PA | Mixed | Qualitative | Interview | PA | Integration and retention of American physician assistants/associates working in English hospitals: A qualitative study |
| [41] | 2018 | US - Maryland | PA | Mixed | Quantitative | Secondary data analysis and survey | N/A | Integration of a Physician Assistant Into an Ophthalmology Consult Service in an Academic Setting |
| [42] | 2019 | Israel | PA | Inpatient | Quantitative | Survey | PA | Integration of the first physician assistants into Israeli emergency departments - The physician assistants' perspective |
| [43] | 2013 | UK - England | PA | Inpatient | Mixed methods | Interview, survey | All clinical staff | Introducing physician assistants into an intensive care unit: process, problems, impact and recommendations |
| [44] | 2007 | US - Kansas | Mixed | Inpatient | Quantitative | Survey | Patient and clinical staff | Midlevel providers in a Level I trauma service: experience at Wesley Medical Center |
| [45] | 2010 | US - Texas | Mixed | Mixed | Qualitative | Reflection | N/A | Multidisciplinary cancer care: Development of an infectious diseases physician assistant workforce at a comprehensive cancer center |
| [46] | 2018 | US - Nationwide | PA | Mixed | Quantitative | Survey | PA | National Study of Burnout and Career Satisfaction Among Physician Assistants in Oncology: Implications for Team-Based Care |
| [47] | 2013 | US - Nationwide | Mixed | Mixed | Quantitative | Survey | Program director | Non-physician providers as clinical providers in cystic fibrosis: Survey of US programs |
| [48] | 2011 | Netherlands | Mixed | Mixed | Mixed methods | Survey and interview | PAs and NPs | Nurse practitioners and physician assistants in Dutch hospitals: their role, extent of substitution and facilitators and barriers experienced in the reallocation of tasks |
| [49] | 2019 | US - Nationwide | Mixed | Emergency | Quantitative | Secondary data analysis | N/A | Nurse practitioners and physician assistants in emergency medical services who billed independently, 2012-2016 |
| [50] | 2002 | US - Nationwide | Mixed | Ambulatory | Quantitative | Secondary data analysis | N/A | Nurse practitioners and physician assistants revisited: do their practice patterns differ in ambulatory care? |
| [51] | 2023 | US - California | Mixed | Mixed | Qualitative | Reflection | N/A | Optimal Use of Advanced Practice Providers at an Academic Medical Center: A First-Year Retrospective Review |
| [52] | 2023 | Ireland | PA | Mixed | Qualitative | Reflection | N/A | PAs in the Republic of Ireland |
| [53] | 2023 | UK - England | PA | Inpatient | Mixed methods | Interview and survey | PA, clinicians, patients | Patient care, integration and collaboration of physician associates in multiprofessional teams: A mixed methods study |
| [54] | 2010 | US - Pennsylvania & Texas | Mixed | Emergency | Quantitative | Survey | Patients, residents and PA | Patient Willingness to Be Seen by Physician Assistants, Nurse Practitioners, and Residents in the Emergency Department: Does the Presumption of Assent Have an Empirical Basis? |
| [55] | 2022 | US - Nationwide | Mixed | Inpatient | Quantitative | Survey | Unit leaders | Patterns of utilization and evaluation of advanced practice providers on hospital medicine teams at academic medical centers |
| [56] | 2020 | UK - England | PA | Inpatient | Qualitative | Interview, document analysis | Doctors, nurses, management | Perceived impact on efficiency and safety of experienced American physician assistants/associates in acute hospital care in England: findings from a multi-site case organisational study |
| [57] | 2012 | US - Connecticut | Mixed | Mixed | Quantitative | Survey | NP and PA | Perceptions of Roles, Practice Patterns, and Professional Growth Opportunities |
| [58] | 2016 | US - Washington | Mixed | Emergency | Mixed methods | Survey and interview | PA, NP, doctors, administrators | Physician Assistants and Nurse Practitioners in Rural Washington Emergency Departments |
| [59] | 2005 | US - New York | PA | Inpatient | Qualitative | Reflection | N/A | Physician assistants as physician extenders in the pediatric intensive care unit setting - A 5-year experience |
| [60] | 2011 | US - Nationwide | PA | Mixed | Quantitative | Survey | PA | Physician assistants in orthopedic practice: A national study |
| [61] | 2010 | US - Nationwide | PA | Mixed | Quantitative | Secondary data analysis | N/A | Physician assistants working in the Department of Veterans Affairs |
| [62] | 2011 | Australia - South Australia | PA | Mixed | Qualitative | Reflection | N/A | Physician assistants: employing a new health provider in the South Australian health system |
| [63] | 2017 | UK - England | PA | Mixed | Quantitative | Survey | Medical directors | Physician associates in England's hospitals: a survey of medical directors exploring current usage and factors affecting recruitment |
| [64] | 2017 | UK - England | PA | Mixed | Quantitative | Survey | PA | Physician associates working in secondary care teams in England: Interprofessional implications from a national survey |
| [65] | 2011 | Australia - Queensland | PA | Mixed | Mixed methods | Survey, interviews, document review | PA, patients, other clinical staff | Physician's assistants: A workforce solution for Australia? |
| [66] | 2005 | US - Georgia | PA | Inpatient | Mixed methods | Reflection | N/A | Physicians assistants in cardiothoracic surgery: A 30-year experience in a university center |
| [67] | 2015 | US - Nationwide | Mixed | Mixed | Quantitative | Survey | Managers | Resident Work Hour Changes in Children's Hospitals: Impact on Staffing Patterns and Workforce Needs |
| [68] | 2021 | US - Nationwide | PA | Emergency | Qualitative | Interview | Emergency PA | Resilience, dysfunctional behavior, and sensemaking: The experiences of emergency medicine physician assistants encountering workplace incivility |
| [69] | 2014 | US - Virgin Island | PA | Mixed | Mixed methods | Observation, interviews | PA, doctors, admin, patients | Role of physician assistants in rural hospital settings in the Virgin Islands: A case study |
| [70] | 2014 | US - Nationwide | PA | Emergency | Quantitative | Survey | PA | Scope of practice and autonomy of physician assistants in rural vs. Urban emergency departments |
| [71] | 2014 | US - Nationwide | PA | Emergency | Quantitative | Secondary data analysis | N/A | State laws governing physician assistant practice in the United States and the impact on emergency medicine |
| [72] | 2020 | US - Texas | Mixed | Inpatient | Mixed methods | Reflection | N/A | Successful Integration of Advanced Practice Providers Into a Pediatric Academic Community Intensive Care Unit |
| [73] | 2009 | Canada - Ontario | Mixed | Emergency | Mixed methods | QI including focus group and survey | PA, ACNP, NP, doctor | The application of change management principles to facilitate the introduction of nurse practitioners and physician assistants into six Ontario emergency departments |
| [74] | 2019 | UK - England | PA | Mixed | Qualitative | Survey | PA | The career aspirations and expectations of student physician associates in the UK |
| [75] | 2021 | US - Nationwide | Mixed | Mixed | Quantitative | Secondary data analysis | PA, NP | The changing landscape of nephrology physician assistants and nurse practitioners |
| [76] | 2021 | US - Tennessee | PA | Mixed | Quantitative | Secondary data analysis | N/A | The Cost of Not Training a Surgical Resident |
| [77] | 2019 | US - Massachusetts | PA | Emergency | Qualitative | Reflection | N/A | The Design and Implementation of a Professional Development Program for Physician Assistants in an Academic Emergency Department |
| [78] | 2006 | US - Nationwide | PA | Inpatient | Quantitative | Survey | PA | The effects of resident work hour restrictions on physician assistant hospital utilization |
| [79] | 2023 | US - California | Mixed | Mixed | Quantitative | Secondary data analysis | N/A | The impact of a structured onboarding program for newly hired nurse practitioners and physician assistants |
| [80] | 2020 | US - Nationwide | Mixed | Emergency | Quantitative | Secondary data analysis | N/A | The Impact of Advanced Practice Provider Staffing on Emergency Department Care: Productivity, Flow, Safety, and Experience |
| [81] | 2017 | Netherlands | PA | Inpatient | Quantitative | Trial | N/A | The impact of the implementation of physician assistants in inpatient care: A multicenter matched-controlled study |
| [82] | 2017 | Netherlands | PA | Inpatient | Quantitative | Economic evaluation | N/A | The involvement of physician assistants in inpatient care in hospitals in the Netherlands: a cost-effectiveness analysis |
| [83] | 2016 | US - Nationwide | Mixed | Outpatient | Quantitative | Secondary data analysis | N/A | The National Ambulatory Medical Care Survey: PAs and NPs in outpatient surgery |
| [84] | 2012 | Canada - British Columbia | PA | Emergency | Mixed methods | Secondary data analysis, survey, focus group discussions | Doctor, PA | The role of physician assistants in a pediatric emergency department: a center review and survey |
| [85] | 2013 | Canada - Nationwide | PA | Emergency | Quantitative | Survey | Doctors | The role of physician assistants in pediatric emergency medicine: the physician's view |
| [86] | 2019 | UK - England | PA | Mixed | Mixed methods | Review, survey, interview, routine data | PA, doctor, nurse, support staff, manager, patient | The role of physician associates in secondary care: the PA-SCER mixed-methods study |
| [87] | 2010 | US - Washington & Oregon | Mixed | Emergency | Quantitative | Survey | ED manager | The Use of Nurse Practitioners and Physician Assistants in Washington and Oregon Emergency Departments: A Descriptive Study of Current Practice |
| [88] | 2000 | US - Nationwide | Mixed | Mixed | Quantitative | Survey | Neurologist | Training the future neurology workforce |
| [89] | 2003 | US - Nationwide | Mixed | Outpatient | Quantitative | Secondary data analysis | N/A | Trends in care by nonphysician clinicians in the United States |
| [90] | 2009 | US - Nationwide | Mixed | Emergency | Quantitative | Secondary data analysis | N/A | Trends in midlevel provider utilization in emergency departments from 1997 to 2006 |
| [91] | 2020 | Canada - Ontario | PA | Mixed | Quantitative | Interview, document review | PA, doctor, admin, other staff | Understanding health professional role integration in complex adaptive systems: a multiple-case study of physician assistants in Ontario, Canada |
| [92] | 2010 | US - Nationwide | Mixed | Emergency | Quantitative | Secondary data analysis | N/A | Use of midlevel providers in US EDs, 1993 to 2005: implications for the workforce |
| [93] | 2002 | US - Montana | Mixed | Mixed | Quantitative | Survey | Hospital admin | Utilization and scope of practice of nurse practitioners and physician assistants in Montana |
| [94] | 2021 | US - Texas | PA | Emergency | Mixed methods | Survey and interview | Managers | Utilization and Workforce Integration of Physician Assistants |
| [95] | 2009 | UK - England | Mixed | Outpatient | Quantitative | Survey | Patient | What is in a name -- patients' view of the involvement of 'care practitioners' in their operations |
| [96] | 2019 | UK - England | PA | Mixed | Mixed methods | Interview, observation, document review, work log | PA, doctor, nurse, support staff, manager, patient | What is the contribution of physician associates in hospital care in England? A mixed methods, multiple case study |
| [97] | 2023 | US - Nationwide | Mixed | Mixed | Quantitative | Secondary data analysis | N/A | What Is the Geographic Distribution and Density of Orthopaedic Advanced Practice Professionals in Rural Counties? A Large-database Study |
| [98] | 2024 | UK - Nationwide | PA | Mixed | Quantitative | Secondary data analysis | N/A | Predictive modelling of the UK physician associate supply: 2014–2038 |
| [99] | 2023 | Canada - Ontario | PA | Mixed | Quantitative | Survey | Doctor | Physician ratings of physician assistant competencies and their experiences and satisfaction working with physician assistants: Results from the supervising physician survey in Ontario, Canada |
| [100] | 2023 | UK - Nationwide | PA | Mixed | Qualitative | Interview | PA, PA educator | Career Development Needs of Physician Associates in the United Kingdom: A Qualitative Study |
| [101] | 2023 | Netherlands | Mixed | Mixed | Quantitative | Survey | PA and PA training programme | The influence of government policies on the nurse practitioner and physician assistant workforce in the Netherlands, 2000– 2022: a multimethod approach study |
| [102] | 2022 | US - Nationwide | Mixed | Mixed | Quantitative | Secondary data analysis | Neurologist | The Evolving Role of Advanced Practice Providers in Otolaryngology: Improving Patient Access and Patient Satisfaction |
| [103] | 2025 | US - Colorado | Mixed | Mixed | Qualitative | Interview | PA, CNPs, CNSs | Voices from the frontline: Perspectives from certiﬁed nurse practitioners, clinical nurse specialists and physician assistants at an Academic Medical Center |
| [104] | 2023 | UK - England | Mixed | Emergency | Qualitative | Interview | Stakeholders | Non-medical practitioners in the staffing of emergency departments and urgent treatment centres in England: a mixed qualitative methods study of policy implementation |
| [105] | 2024 | US - Nationwide | Mixed | Mixed | Quantitative | Survey | PA, Advanced Practice Nurse | Advanced Practice Providers in Cellular Therapy: Survey Results from the ASTCT APP Special Interest Group Exploring Clinical Roles, Compensation, and Job Satisfaction |
| [106] | 2023 | US - Nationwide | Mixed | Mixed | Quantitative | Survey | PA, APP, fellow, programme directors | Building Relationships: Advanced Practice Providers and Fellows in Neonatal-Perinatal Medicine |
| [107] | 2023 | US - Nationwide | Mixed | Mixed | National level data | Secondary data analysis | N/A | The changing employment of physicians, nurse practitioners, and physician associates/assistants |
| [108] | 2024 | US - Nationwide | Mixed | Mixed | National level data | Secondary data analysis | N/A | Specialization of Physician Associates and Nurse Practitioners as Reflected in Workforce Projections |
| [109] | 2024 | US - Tennessee | Mixed | Mixed | Qualitative | QI | N/A | Integration of a nurse practitioner and physician associate leadership structure within an academic cancer center |
| [110] | 2023 | US - DC | PA | Emergency | Quantitative | Survey | Senior PA and supervisors | Development of a Physician Assistant Orientation Program in a Pediatric Emergency Department |
| [111] | 2023 | South Korea | PA | Mixed | Quantitative | Survey | PA | Job satisfaction and moral distress of nurses working as physician assistants: focusing on moderating role of moral distress in effects of professional identity and work environment on job satisfaction |
| [112] | 2024 | UK - England | PA | Emergency | Quantitative | Secondary data analysis | N/A | Comparing physician associates and foundation year 1 doctors- in- training undertaking emergency medicine consultations in England: a quantitative study of outcomes |
| [113] | 2024 | UK - England | PA | Emergency | Mixed methods | Survey, interviews, document review | PA, consultants, patients | Doctors', Patients' and Physician Associates' Perceptions of the Physician Associate Role in the Emergency Department |
| [114] | 2024 | US - Nationwide | Mixed | Emergency | Quantitative | Secondary data analysis | N/A | Utilizing Nurse Practitioners and Physician Assistants in Academic Emergency Departments Does Not Reduce Residents’ Exposure to More Complex Patients |
| [5] | 2024 | US - New York | PA | Mixed | Quantitative | Survey | Doctors, NPs | How Do Physicians and Nurse Practitioners Perceive the Title Change from Physician Assistant to Physician Associate? |
| [115] | 2025 | US - Minnesota | Mixed | Inpatient | Quantitative | Administrative data | N/A | Nurse Practitioner and Physician Assistant-led Cardiovascular Surgery Postoperative Intensive Care Unit Stafﬁng Model |
| [116] | 2023 | UK - England | PA | Mixed | Qualitative | Interview | PA, doctors | Facilitators to the integration of the first UK-educated physician associates into secondary care services in the NHS |
| [117] | 2023 | US - Nationwide | PA | Mixed | Quantitative | Survey | PA | Procedures by Physician Associates in Obstetrics and Gynecology |
| [118] | 2024 | US - Nationwide | PA | Mixed | Quantitative | Secondary data analysis | N/A | Demographics of Physician Associates (PAs) in Obstetrics and Gynecology: Where They Work and How They Compare to Other PAs |
| [119] | 2025 | US - Nationwide | PA | Mixed | Quantitative | Survey | PA | The Impact of the COVID-19 Pandemic on Work-Life Integration of Physician Assistants in Oncology |
| [120] | 2024 | US - Nationwide | PA | Mixed | Qualitative | Policy review | N/A | Advanced practice providers: An evolution of scope of practice and clinical integration across the surgical healthcare landscape |
| [121] | 2023 | US - Nationwide | PA | Mixed | Quantitative | Survey | PA | A National Survey of Perspectives of Physician Assistants in Academic Plastic and Reconstructive Surgery |
| [122] | 2019 | UK - Nationwide | PA | Mixed | Quantitative | Survey | Healthcare professionals | The Regulation of Medical Associate Professions in the UK |
| [123] | 2024 | UK - Nationwide | PA | Mixed | Qualitative | Consultation | N/A | Consultation outcome Regulating anaesthesia associates and physician associates |
| [124] | 2025 | UK - Nationwide | PA | Mixed | Quantitative | Administrative data | N/A | GMC response to the Leng Review’s call for evidence |
| [125] | 2024 | UK - Nationwide | PA | Mixed | Qualitative | Interview, focus group | Public | Exploring public views on the implementation of regulatory reform |
| [126] | 2024 | UK - Nationwide | PA | Mixed | Qualitative | Consultation | N/A | Regulating physician associates and anaesthesia associates Proposed rules, standards and guidance: report on the public consultation |
| [127] | 2025 | UK - Nationwide | PA | Mixed | Qualitative | Court document | N/A | British Medical Association -v- General Medical Council |
| [128] | 2023 | UK - Nationwide | PA | Mixed | Quantitative | Survey | Public | Public omnibus survey – PAs and AAs – |
| [129] | 2024 | UK - Nationwide | PA | Mixed | Quantitative | Survey | Doctor | BMA Medical Associate Professions (MAPs) survey |
| [130] | 2025 | UK - Nationwide | PA | Mixed | Quantitative | Survey | Doctor | BMA member survey on PAs, AAs and safety. |
| [22] | 2025 | UK - Nationwide | PA | Mixed | Qualitative | Consultation | Doctor | BMA Reporting Portal Submissions Physician Associates and Anaesthesia Associates |
| [131] | 2025 | UK - Nationwide | PA | Mixed | Qualitative | Consultation | Doctor | BMA submission to the independent review of the physician associate and anaesthesia associate professions |
| [132] | 2025 | UK - Nationwide | PA | Mixed | Qualitative | Consultation | Doctor | Doctors’ Association UK - Submission to the Leng Review on MAPs |
| [133] | 2024 | UK - Nationwide | PA | Mixed | Qualitative | Reports | PA, doctor | The physician associates becoming doctors |
| [134] | 2018 | UK - Nationwide | PA | Mixed | Qualitative | Reports | Doctor | Medical associate professions: how physician associate and similar roles are developing, and what that means for doctors |
| [135] | 2025 | UK - Nationwide | PA | Mixed | Qualitative | Consultation | Doctor | RCP response to the independent review of physician associate and anaesthesia associate professions |
| [136] | 2024 | UK - Nationwide | PA | Mixed | Quantitative | Survey | Doctor | Research Report Royal College of Paediatrics and Child Health Physician Associates Member Consultation |
| [137] | 2024 | UK - Nationwide | PA | Mixed | Qualitative | Survey | Doctor | Research Report Royal College of Paediatrics and Child Health Physician Associates Member Consultation Further Data Analysis Opinions |
| [138] | 2024 | UK - Nationwide | PA | Mixed | Qualitative | Survey | Doctor | Research Report Royal College of Paediatrics and Child Health Physician Associates Member Consultation Further Data Analysis Paediatric Experiences and Opinions by Region, Frequency & Workplace |
| [139] | 2024 | UK - Nationwide | PA | Mixed | Qualitative | Survey | Doctor | Research Report Royal College of Paediatrics and Child Health Physician Associates Member Consultation Further Data Analysis Supervision, training and signoff |
| [140] | 2025 | UK - Nationwide | PA | Mixed | Qualitative | Consultation | Doctor | Physician Associates - RCPCH response to member consultation |
| [141] | 2024 | UK - Nationwide | PA | Mixed | Quantitative | Survey | Doctor | Physician Associates in Paediatrics |
| [142] | 2025 | UK - Nationwide | PA | Mixed | Quantitative | Consultation | PA, doctor | Summary Report for the Leng Review: Evaluating Physician Associate Colleague Multi-Source Feedback |
