# Supplementary material for: Developing and integrating physician assistants/associates in UK hospital teams: a realist review of lessons from international experiences
Source: BMC Med. 2025 Dec 29;23:707. doi: 10.1186/s12916-025-04530-z (PMC12751915; doi:10.1186/s12916-025-04530-z)
Supplement: Supplementary file 4 — Additional file 4. Background information of the nine countries and territories including states and provinces that govern PA practices separately. [file 12916_2025_4530_MOESM4_ESM.docx]

**Additional File 4. Background information of the nine countries and territories including states and provinces that govern PA practices separately**

| Country | Number of PA | PA to population ratio (per 100,000) | Year of first PA graduation | Number of PA programs | Policy and legislation overview | Prescriptive authority |
| --- | --- | --- | --- | --- | --- | --- |
| US | 145,740 | 44 | 1967 | 310 | Federal acts and polices (18 through 1966 and 2024) provided educational and service payment support for PA to provide services, e.g. Medicare coverage of services provided by PAs in certified rural health clinics was first authorised in 1977 and gradually expanded to cover all settings in 1997 for Medicare and Medicaid; State law and licensure boards further govern PA practices, most states recognised PA between 1970-1980 with late adopters such as Mississippi (which took 30 years to pass legislation due to nurse practitioners resistance), however huge variations in terms of scope of practice, physician supervision such as co-signature and chart review | Yes, prescribing in all 50 states and DC and Guam |
| US – California | 12,880 | 34 *(Ranked 45 out of 49 states)* | 1974 | 21 | Initial recognition in 1971; restriction on surgical procedure and emergency provision; minimum daily supervision (available by telecommunication), co-signature required, max PA ratio 2 or 4 (in underserved areas) | Yes, allow schedule II medications, prescription review not required |
| US – Connecticut | 2,950 | 83 *(Ranked 3 out of 49 states)* | 1975 | 6 | Initial state recognition in 1980; minimum daily supervision (available by), co-signature required, max PA ratio 6 | Yes, allow schedule II medications, prescription review required |
| US – Florida | 9,060 | 50 *(Ranked 28 out of 49 states)* | 1973 | 16 | Initial recognition in 1976; restriction on surgical procedure, anaesthesia, emergency provision; minimum daily supervision (available by telecommunication), co-signature not required, max PA ratio 4 | Yes, prescription review required |
| US – Georgia | 3,760 | 43 *(Ranked 37 out of 49 states)* | 1972 | 6 | Initial recognition in 1972; restriction on emergency provision; minimum daily supervision (available by telecommunication), co-signature required, max PA ratio 2 | Yes, prescription review required |
| US – Kansas | 800 | 47 *(Ranked 32 out of 49 states)* | 1973 | 2 | Initial recognition in 1973; restriction on emergency provision; minimum daily supervision (available in-person), co-signature required, max PA ratio 2 | Yes, allow schedule II medications, prescription review not required |
| US – Maryland | 3,280 | 59 *(Ranked 20 out of 49 states)* | 1972 | 5 | Initial recognition in 1972; restriction on emergency provision; minimum daily supervision (available by telecommunication), co-signature not required, max PA ratio 2 | Yes, allow schedule II medications, prescription review not required |
| US – Massachusetts | 4,130 | 65 *(Ranked 15 out of 49 states)* | 1972 | 8 | Initial recognition in 1973; restriction on surgical procedure, anaesthesia, emergency provision; minimum daily supervision not specified, co-signature required, max PA ratio 2 | Yes, allow schedule II medications, prescription review not required |
| US – Montana | 660 | 79 *(Ranked 5 out of 49 states)* | 1996 | 1 | Initial recognition in 1981; minimum daily supervision (available by telecommunication), co-signature required, max PA ratio not specified | Yes, allow schedule II medications, prescription review not required |
| US – New York | 16,690 | 79 *(Ranked 6 out of 49 states)* | 1972 | 29 | Initial recognition in 1972; minimum daily supervision not specified, co-signature required, max PA ratio 6 | Yes, allow schedule II medications, prescription review not required |
| US – Oregon | 1,420 | 55 *(Ranked 22 out of 49 states)* | 1996 | 3 | Initial recognition in 1971; minimum daily supervision (available by telecommunication), co-signature not required, max PA ratio 4 | Yes, allow schedule II medications, prescription review not required |
| US – Pennsylvania | 8,730 | 85 *(Ranked 2 out of 49 states)* | 1972 | 31 | Initial recognition in 1978; restriction on surgical procedure, anaesthesia, emergency provision; minimum daily supervision (available by telecommunication), co-signature required, max PA ratio 4 | Yes, allow schedule II medications, prescription review required |
| US – Tennessee | 2,240 | 43 *(Ranked 38 out of 49 states)* | 1978 | 12 | Initial recognition in 1980; restriction on surgical procedure, emergency provision; minimum daily supervision not specified, co-signature required, max PA ratio not specified | Yes, allow schedule II medications, prescription review not required |
| US – Texas | 8,630 | 37 *(Ranked 42 out of 49 states)* | 1973 | 14 | Initial recognition in 1981; minimum daily supervision (available by telecommunication), co-signature required, max PA ratio 3 | Yes, prescription review required and for refill only |
| US – Virginia | 3,760 | 52 *(Ranked 23 out of 49 states)* | 1997 | 9 | Initial recognition in 1973; restriction on surgical procedure, emergency provision; minimum daily supervision (available in-person), co-signature required, max PA ratio 2 | Yes, allow schedule II medications, prescription review not required |
| US – Washington | 3,240 | 51 *(Ranked 24 out of 49 states)* | 1973 | 2 | Initial recognition in 1971; minimum daily supervision not specified, co-signature required, max PA ratio 4 | Yes, allow schedule II medications, prescription review not required |
| US – Virgin Islands | 30 | 28 | N/A | N/A | Initial recognition in 2005; PA must be continuously supervised, max PA ratio 3 | No |
| UK | 3,440 | 5 | 2004 | 37 | The UK and Scottish parliaments have approved legislation that allow the General Medical Council to regulate PAs from Dec 2024, standards and guidance in relation to scope of practice and supervision are under preparation | No |
| UK – England | 3,286 | 6 | 2004 | 36 | PA role is recognised in the NHS Long Term Workforce Plan published in June 2023 which aims to increase the workforce to 10,000 by 2036-37 | No |
| Ireland | 76 | 1 | 2017 | 1 | Not yet regulated and PA now operate under "delegated autonomy" | No |
| Taiwan | 0 | 0 | N/A | N/A | Efforts in 2005 to formalise the education process and legalise the PA profession met resistance from the medical and nursing profession, therefore all PAs became nurse practitioners in 2005 | No |
| Canada | 959 | 3 | 2005 | 4 | Medical services and professions regulation and billing requirement at provincial level, and seven of 10 provinces and three territories have established or currently establishing formal PA regulations, especially over the past five years | Yes, in selected provinces |
| Canada - Manitoba | 142 | 11 | 2008 | 1 | Initial recognition in 1991 as clinical assistant, the first in Canada; scope of practice and supervision arrangement determined by the contract of supervision and practice description; service cannot be billed and reimbursed directly by the PA | Yes, in accordance to their contract of supervision, allow M3P medications with additional requirement |
| Canada - Ontario | 703 | 4 | 2008 | 2 | The government current uses the Delegated Acts provision of the Ontario Medical Act, and official regulation expected April 2025; supervision through either approval by physicians in writing or verbally required for each act performed by the PA, or through the use of a medical directive | Yes, via delegation of controlled acts |
| Canada - British Columbia | 32 | 1 | N/A | N/A | Not yet regulated, bylaw expected to permit PAs to practice in emergency departments; supervision required as all work to be assigned, supervised or delegated by the supervision physician | No |
| Netherlands | 1,590 | 9 | 2003 | 5 | National full recognition and practice authority established through a 2012 provisional law that was codified in 2018; within a clearly defined and negotiated scope of practice, physician supervision is not obligatory, even though a collaborative partnership with a physician is encouraged | Yes, full prescription authority |
| Australia | 30 | 0.1 | 2011 | 1 | At federal level, PA roles recognised by Health Workforce Australia in 2011, however service reimbursement from Medicare and Pharmaceutical Benefits Schedule not recognised yet; At state level, only one of 6 states and 10 federal territories (Queensland) has legislation | Yes, in selected states |
| Australia – Queensland | N/A | N/A | 2011 | 1 | Initial regulation in 2021; PA practice under delegation and supervision, which is determined based on clinical type, patient acuity, healthcare setting and PA competence, this could be either direct supervision or indirect supervision defined in the practice plan; max PA ratio 2; scope of practice evolve | Yes, under supervision and within practice plan |
| Australia – South Australia | N/A | N/A | N/A | N/A | Not yet regulated | Yes, in accordance to state act and through state-level negotiations |
| Israel | 100 | 1 | 2017 | 1 | Legislation to regulate PAs approved in 2023; exclusively in emergency departments; scope of practice and supervision arrangement determined by specialist physicians | Yes, in emergency department only |
| South Korea | 6,000 | 12 | N/A | N/A | Under the Nursing Act to allow nurses to work as PAs | No |

*Note: All number and ratio are data from 2022-2024, aside from Australia which is from 2020*
