# Supplementary material for: Developing and integrating physician assistants/associates in UK hospital teams: a realist review of lessons from international experiences
Source: BMC Med. 2025 Dec 29;23:707. doi: 10.1186/s12916-025-04530-z (PMC12751915; doi:10.1186/s12916-025-04530-z)
Supplement: Supplementary file 5 — Additional file 5. List of CMOC, representative quotes, and supporting data. [file 12916_2025_4530_MOESM5_ESM.docx]

**Additional File 5. List of CMOC, representative quotes, and supporting data**

| **CMOC** | **Representative quotes** | **Supporting data** |
| --- | --- | --- |
| **ORGANISATIONAL DRIVER** | | |
| **CMOC A1: When hospital and department leaders perceive challenges in service delivery (C), they are more likely to consider changes to workforce models (O) because they are dissatisfied with the status quo or pressured to resolve the challenge (M).** | - Necessity, in order to address four problems, was the most commonly cited reason for beginning to employ PAs…Some participants described the problems particularly in relation to shortages of doctors to meet service requirements as ‘a crisis’ or a ‘burning platform’. Many participants discussed redesign of their workforce as an ongoing process. *(*[86]*– UK England)* - Workload, educational, and ﬁnancial considerations drove the decision to incorporate MLPs into the surgical service, and the decision was reinforced by the passage of the Accreditation Council for Graduate Medical Education 80-hour workweek. Decrease in total and consecutive allowable hours resulted in a personnel deﬁcit, which potentially threatened both provision of care and resident physician education. MLPs provided a win-win solution... *(*[40]*– US Virginia)* - Signiﬁcant medical workforce shortages, particularly in rural and remote locations, have prompted a range of responses in Australia at both state and Commonwealth levels. One such response was a pilot project to test the suitability of the Physician Assistant (PA) role in the Australian context. *(*[65]*– Australia Queensland)* | N=27  [24, 31, 32, 34, 35, 37, 39, 41, 44, 45, 47, 49, 56, 59, 61, 65–67, 73, 78, 84, 86, 87, 96, 101, 104, 134] |
| **CMOC A2: When hospital and department leaders are satisfied with existing workforce model or cannot see a clear need for change (C), they are unlikely to invest in developing PA roles (O) because they do not perceive PAs ad offering additional value (M).** | - An important factor for not employing a PA is that there is no need for change because of satisfaction with the current quality of care and the availability of appropriate residents. *(*[32]*- Netherlands)* - Administrators feeling as though the workload was not enough in every department to support a PA, a physician, or physicians. *(*[69]*- US - Virgin Island)* - However, there was also uncertainty and differing views amongst participants. Divergent views were offered as to whether there was publicly available evidence of clinical safety, efficiency, and cost effectiveness i.e., relative advantage of having NMPs in EDs/UTCs. *(*[104]*– UK England)* | N=8  [25, 32, 41, 43, 55, 69, 84, 104] |
| **CMOC A3: When there is external policy guidance, political and financial support for PA roles (C), hospital and department leaders are more likely to engage with PA role development (O) because they feel legitimised to do so (M)** | - The Department of Health (DoH) commissioned an evaluation of the pilot led by the Changing Workforce Programme (CWP), which resulted in recommendations to introduce the PA role more widely in the National Health Service (NHS), with a preference for UK-recruited and trained individuals. *(*[31]*– UK England)* - Statutory regulation would give employers greater confidence in employing MAPs therefore current costs associated with employing agency or locum staff would be reduced. *(*[122]*– UK Nationwide)* - The Balanced Budget Act of 1997 advanced the economic incentive for hospitals to hire PAs and NPs when it made their hospital services eligible for Medicare reimbursement at 85% of the physician rate. *(*[40]*– US Virginia)* | N=23  [25, 27, 31, 32, 39, 40, 42, 48, 52, 62, 63, 69, 86, 98–102, 104, 107, 122, 123, 140] |
| **CMOC A4: When hospital and department leaders and individual clinical team members have previous positive experience with PA roles or local experience to observe (C), they may be more willing to invest in and accept PA roles (O), because these experiences can influence perception of potential contribution (M).** | - Of the sample surveyed, 92% of respondents were already familiar with the PA role, with the most common reason for the familiarity being the employment of a PA on the team. The next most common reason for being familiar with the role was their training experience in North America or from a colleague who had a PA on their team. *(*[25]*- Ireland)* - Positive experiences with PAs seem to positively stimulate the employment of PAs… Experiencing positive outcomes of PAs positively inﬂuence the sustainability of the implementation of PAs in inpatient care. *(*[32]*- Netherlands)* - These interactions built up a level of comfort and trust that enabled the ID department to incorporate PAs and NPs into its workforce... the ID department would strongly recommend PAs or NPs to other disciplines within the institution*. (*[45]*– US Texas)* | N=15  [25, 32, 38, 41, 45, 52, 63, 69, 73, 85, 86, 99, 104, 113, 137] |
| **CMOC A5: When there is external organised professional resistance against employing PAs (C), hospital and department leaders are less likely to do so (O) because such resistance can reduce organisational confidence for developing the role (M)** | - There is strong opposition to the role in Australia from a broad cross section of health professions including the medical and nursing professions. While the role is promoted as a solution to workforce shortages, the root cause of these shortages is the chronic failure to invest properly in the existing medical workforce and the broader health system. It is disappointing that policy makers ignore this and look instead to simplistic solutions that fragment care, deliver poorer health care outcomes and result in higher costs in the longer term. *(*[131]*– UK Nationwide)* - When the new role was introduced, there was a fair amount of uncertainty about whether it would succeed, in light of strong resistance from various elements of the emergency medicine scene in Israel. The first obstacle was a resolute objection to the new position by the nurse’s union, due to the threat that this position imposes on the nurses’ position and authority in the ED.…. Another strong opponent of this initiative was the Israeli emergency medicine organization Magen David Adom. *(*[42]*– Israel)* - Resistance from professional associations of medical specialists was mentioned as a factor which inﬂuences the decision to not employ a PA. *(*[32]*- Netherlands)* | N=6  [25, 32, 42, 63, 104, 131] |
| **CMOC A6: When hospitals and department leaders have internal competing priorities or interests in terms of workforce planning (C), they may deprioritise PA role development (O) because they prioritise roles that align with their professional objectives (M).** | - Although most of the doctors reported that nurses, particularly at the ward level, were positive about PAs, some negative views and points of tension were also reported. Some senior nurses were reported to actively block their employment and development, preferring to support ANPs: She’s [chief nurse] the person who has the worst opinion of our physician associate role . . . her ambition is for more ANPs . . . she wants all development monies to go there. *(*[86]*– UK England)* - The authors have anecdotally heard that nursing leadership has reignited opposition to the PA role since the conclusion of this research project which may be linked to the nursing initiative to advance a nurse practitioner role. *(*[42]*– Israel)* - While concerns diminished regarding the relationship of the PA role to the junior doctor position, concerns did remain regarding health system capacity, given the increasing number of medical students expected to graduate in the next few years, and the corresponding increase in the number of junior doctors seeking training places… The second concern was that establishing the PA role may reduce opportunities for nurse practitioners (NPs) and other expanded nursing roles. *(*[65]*– Australia Queensland)* | N=4  [42, 66, 73, 86] |
| **CMOC A7: When there is a lack of organisational workforce long-term planning and vision (C), role development become challenging (O) because there is no clear direction, commitment or resource to support these role (M)** | - These organizational and policy problems predominantly included lack of long-term planning and vision by the management/board of directors, resistance from medical specialists or junior doctors, insecurity about continuation or job content, unclear position in the organization. *(*[48]*- Netherlands)* - Many participants pointed to the negative impact of the lack of national, regional and often local level health care workforce plans. “The NHS only seems to really plan for the next 12 months from April to April, and we really struggle to persuade organisations that, actually, this is part of a five-year, ten-year plan, and they’ve got to invest now [in ACP in ED training] in order to have that positive effect much later on down the line”. *(*[104]*– UK England)* - Occasionally, there was a perception that clarity about what was required had not been fully achieved and that PAs had been ‘dumped in’ and then problems had to be sorted out. *(*[86]*– UK England)* | N=6  [32, 48, 56, 62, 86, 104] |
| **CMOC A8: When a specific and accessible funding source for PA roles exists (C),** **hospital and department leaders are more likely to create PA roles (O) because it makes financial sense to do so (M).** | - Identification of a funding stream for the PA is essential to encourage engagement and employment at hospital level. *(*[25]*– Ireland)* - Starting in November 2011, the Infectious Diseases Consult Service (IDCS) at Toronto East General Hospital (Toronto, Ontario), a 515bed urban community hospital, introduced a PA with assistance from a Career Start Grant for Physician Assistant Graduates through Health Force Ontario *(*[38]*– Canada Ontario)* - Students were hired by a specific emergency department before beginning the course, each ED had an option to hire between one and three PA trainees. The MOH pilot program funded each participant’s salary during the yearlong training. *(*[42]*– Israel)* | N=9  [25, 34, 38, 42, 51, 59, 91, 99, 104] |
| **CMOC A9: When there is uncertainty and disagreement regarding the funding source for PA roles (C), hospital and department leaders may be less committed to PA role development (O) because financial ambiguity and competition makes it unclear whether to prioritise and sustain the role (M).** | - Financial problems were experienced by more than half of the NPs (53.5%; n = 23) and PAs (53.8%; n = 7). Financial problems mainly concerned uncertainty about the budget (medical/nursing) to fund the employment of NPs or PAs, whether the medical partnership had to or was willing to contribute to the salaries of NPs and PAs, and the salary levels of NPs and PAs. *(*[48]*– Netherlands)* - Funding comes from multiple sources, including global hospital budgets, departments, pay-for-performance, other allocated funding sources (i.e., Family Health Team allied health funds) or directly from physicians. The challenge of these variable sources is the dependency on intermittent, short stream funding and its impact on role sustainability. *(*[91]*– Canada Ontario)* - *[PA]* posts in the hospital are not being funded by, at senior level so they have to be employed on the basis of individual departments, which obviously makes it a lot more challenging trying to get them into the workplace. *(*[53]*– UK England)* | N=11  [25, 32, 47, 48, 53, 86, 91, 99, 101, 104, 135] |
| **CMOC A10: When there is sufficient and locally accessible PA supply that meets hospital and department needs, including those with specialised training (C),** **hospital and department leaders are more likely to invest in developing PA roles (O), because it would be relatively easy to recruit and fill these positions (M)** | - All sites were planning to employ NQPAs during the study period in the context of a recent expansion in student PA numbers within the region. *(*[23]*– UK England)* - Our research ﬁndings highlight an increased awareness regarding the lack of available PAs to hire…Increased interest and uptake of PAs across the province has created an imbalance between the supply (low number of graduates) and demand (high number of positions), thus making it more challenging for physician employers to add additional PAs to their team. *(*[99]*– Canada Ontario)* - Where there were previous posts filled by a PA who subsequently left the post (n = 4), the reason for three of these posts remaining unfilled was the unsuccessful employment of a PA, relating back to the low numbers of graduates to date. *(*[25]*- Ireland)* | N=19  [23, 25, 31, 40, 41, 49, 63, 67, 69, 78, 86, 87, 98–101, 106, 109, 113] |
| **CMOC A11: When hospital and department leaders can make a compelling case for committing resources to develop and integrate PAs (C), this is more likely to occur (O) because decision-makers are more likely to be convinced of its value (M)** | - Appealing to the value of patient safety and efﬁciency works with administrators…Table 1 lists an example of “running the numbers” for administration. Section A provides the administrator with clear quantiﬁcation of the person-hours lost under the 80hour workweek rule. Section B identiﬁes minimal coverage requirements for safe patient care and deﬁcits that may now exist. This deﬁcit number can be converted to FTE and used to support the addition of MLPs. *(*[40]*– US Virginia)* - However, a decrease in LOS by 3.6 days per patient would undoubtedly be associated with significant cost savings when the cost per day of hospital admission is considered and could form the basis of a business plan to justify employing a PA. *(*[38]*– Canada Ontario)* - The desired continuity of care appeared to be a main deciding factor for the employment of PAs in inpatient care… PAs generally do not rotate and can be a more stable factor in the continually changing medical workforce, which is thought to lead to advantages in quality of care. *(*[32]*- Netherlands)* | N=11  [32, 37–39, 41, 43, 51, 69, 78, 79, 107] |
| **CMOC A12: When there is an authoritative, capable and well-supported champion for PAs (C), PA roles are more likely to be institutionalised and sustained (O) because champions are willing to devote time and energy to drive advocacy, engagement and implementation (M)** | - PAs have a champion who is willing and able to provide support and advocacy…Consultants felt that it was their duty to model acceptance and advocacy for their PAs to their junior doctors. *(*[116]*– UK England)* - Physician champions need to be engaged from the very beginning as these early adopters are key to overall acceptance. *(*[73]*– Canada Ontario)* - In other cases, clinical leaders had been identified as PA champions, promoting the role within the organisation and encouraging colleague engagement. This resulted in positive outcomes; PA (5) I think from the very start of us starting here, the consultants and the [clinical] lead they were very supportive and they were aware of the things that were going through our mind and trying to reassure us. *(*[23]*– UK England)* | N=11  [23, 32, 37, 39, 43, 51, 52, 73, 86, 104, 116] |
| **ROLE AND IDENTITY FORMATION** | | |
| **CMOC B1:** **When decision-making is decentralised, and departments or clinical teams have the authority to determine staffing and roles (C), PA roles may be tailored to meet local service demands (****such as taking on specialised tasks or contributing to team-based working) (O) because department leaders believe they can use their authority to adapt the role as they see fit (M).** | - The first PA cardiology post in the UK, the position was salaried, providing service with direct impact upon patient care. The supervising consultant cardiologist, a teacher on the PA course, was responsible for determining the scope of duties and responsibilities, with overall accountability for the work of the PA, similar to that of junior medical trainees. *(*[31]*- UK England)* - The concept and role of MLPs was thoroughly discussed with house staff in their orientations. Each team provided input on the needs of the team, and the program director selected the candidate with the skill set that best matched those needs. The job description for each MLP was tailored to the service, team, and units on which they work. Thus, each team received what they wanted and needed. *(*[40]*– US Virginia)* - We have a devolved clinical structure here. It’s up to the clinical business units to decide really on the model of care and the resource that they believe that they need to be able to manage that. *(*[86]*– UK England)* | N=20  [23, 29, 31, 32, 39, 40, 45, 53, 56, 59, 66, 73, 86, 91, 96, 102, 103, 113, 115, 124] |
| **CMOC B2: When the roles and responsibilities are not defined locally by hospitals, departments and clinical teams (C), development of PA roles becomes challenging (O) because PAs individual PAs and other clinical team members have confusion about role expectations (M).** | - This qualitative study found that organisational uncertainties about the PA role impacted on the early integration experiences of skilled American PAs in English hospitals. These uncertainties concerned how the PA role was to be deﬁned and delivered as well as how it might affect the roles of other medical/surgical team members. Contributory factors to the uncertainties included staff being naive about the PA role, poor awareness and understanding of the skills and competencies of the PAs, and confusion over how to manage their lack of authority in England to prescribe and order ionising radiation. *(*[40]*– UK England)* - When the new role was introduced, there was a fair amount of uncertainty about whether it would succeed, in light of ambivalence on the part of many ED nurses, and lack of clarity among ED directors about the necessity of a PA role, and about the extent to which PAs would be allowed to take on professionally meaningful tasks. *(*[42]*– Israel)* - The lack of clarity of the PA role and how it fits into the wider multi-professional team appeared to be due to little guidance being provided to staff and patients before the PAs were introduced to the teams. *(*[53]*– UK England)* | N=10  [39, 40, 42, 53, 56, 86, 104, 113, 126, 127] |
| **CMOC B3: When supervision responsibilities and lines of accountability are unclear or inconsistently communicated to team members (C), individual PAs and other clinical team members may experience confusion and uncertainty about their responsibilities (O) because there is ambiguity about clinical authority (M).** | - Many participants, particularly junior doctors reported uncertainty about responsibility for supervising NQPAs potentially inhibiting collaborative working; CT (4): With the physician associates, you don’t know if you should be making decisions for them. Is that my responsibility? If I say yes to something that they do, and that wasn’t right, then was that my fault? *(*[23]*– UK England)* - The “Low Level Interactions” include lines of communication and authority among the mid-level residents, NPPs, and junior residents. The survey results demonstrate that many junior residents feel this is a problem area. They can get mixed messages from the chief resident–mid-level resident pathway on the one hand and the attending–NPP pathway on the other. Again, these relationships should be better deﬁned by the division chiefs, minimizing misunderstanding about roles and authority on the team. *(*[37]*– US Pennsylvania)* - 49% (of RCPCH members) do not know if PAs have a clinical supervisor on shift. *(*[140]*– UK Nationwide)* | N=26  [22, 23, 37, 39, 41, 43, 53, 59, 63, 64, 67, 86, 100, 106, 113, 122, 124, 126, 129–132, 135, 137, 139, 140] |
| **CMOC B4:** **When organisations provide clear communication about PA roles (C), clinical team members better understand what PAs can do (O) because this contributes to clarity and aligns expectation across the team (M).** | - In some trusts, considerable efforts had been made to improve the understanding of PA roles. Activities included: (1) information giving at consultants’ meetings, (2) coaching for the whole team on working with PAs and what they do, (3) explanations of the PA role given to junior doctors during inductions, (4) publicly displayed posters introducing PAs, (5) promotion of sources of information about PAs. *(*[86]*– UK England)* - The role of the PAs and their programme were discussed widely and openly before their introduction within PICU, but there was a lack of clear and accessible information. Simple posters stating the purpose, capabilities and team role of PAs might have cleared up early misapprehensions. *(*[43]*– UK England)* - Before the two PAs arrived at QEH, the hospital formed a trial-site implementation group that consisted of interested parties from the hospital. They included medical, nursing, allied health, human resources and administration staff, as well as representatives of the Royal Australasian College of Surgeons… The important functions of these meetings were to educate the implementation group, address the group’s concerns, and create an environment where the trial was viewed as a trial rather than a turf war. *(*[62]*– Australia South Australia)* | N=18  [23, 31, 37, 39, 40, 43, 51, 53, 62, 65, 73, 74, 79, 86, 106, 109, 130, 135] |
| **CMOC B5:** **When clinical team members have high staff turnover or frequently change (C), regular communications about PAs’ role are needed (O), because**  **gaps in team continuity lead to loss of shared understanding about PA roles (M).** | - In general surgery and other inpatient settings, role clarity is more complicated due to turnover of residents, patients, surgeons/staff physicians in the midst of new consults, discharges and larger interprofessional healthcare teams. *(*[91]*– Canada Ontario)* - However, a few participants described working all the time with different doctors. They reported it was therefore difﬁcult to achieve a level of trust in the working relationship where they felt able to utilise their experience. *(*[40]*– UK England)* - For the most part, the interviewees reported not so much outright opposition as ‘puzzlement’ and general lack of understanding of what PAs were and what they could do. To some extent, this was also reported to be more of an ongoing issue among doctors in training as they rotated from one hospital to another and had to become familiar with PAs. *(*[86]*– UK England)* | N=4  [37, 40, 86, 91] |
| **CMOC B6: When supervisors are supportive and actively guide PAs through workplace hierarchy and role boundaries (C), they will facilitate the PAs’ role development (O) because this support and guidance enable the PA to navigate team relationships and create development opportunities (M).** | - Many participants identiﬁed the key role of dedicated supervisors in promoting integration, particularly in acting as an advocate, facilitating training and planning career development. *(*[23]*– UK England)* - The importance of a supportive supervisor for facilitating the PA’s integration into their team was also mentioned by several participants. “Sometimes it’s a little sensitive making sure that I’m not stepping on the junior doctors’ toes . . .What I think is good is that my consultant doctor is aware of that, so kind of outlined my job plans so that my work wouldn’t directly interfere with the junior doctors’ learning.” (Participant 11) *(*[40]*– UK England)* - In the current study, most respondents were generally satisﬁed with their jobs, but there was a strong relationship between job satisfaction and the PA’s satisfaction with his or her ability to communicate with her coworkers, suggesting that when PAs need good communication with other members of their department, particularly the supervising physician, they can resolve these conﬂicts and experience a higher level of job satisfaction. *(*[30]*– Taiwan)* | N=10  [23, 30, 31, 40, 46, 53, 100, 103, 124, 126] |
| **CMOC B7: When PAs do not receive regular supervision or appraisals (C), they feel uncertain about their role and identity (O) because the lack of feedback hinders role development (M).** | - I haven't had an appraisal, no one has actually sat with us and kind of decided where things are going and that is quite scary and then also there is no feedback, and because of that there is no feedback I don't really know how I am doing. *(*[53]*– UK England)* - “The main guy that I’ve been working with [. . .] had a rough plan, a rough outline of what my job role would be, which was really great because it at least gave me an idea of what they were expecting of me, and then I was able to take that and really help deﬁne the role.” *(*[40]*– UK England)* - Sincere recognition for work done is an obvious means to facilitate a feeling of being valued. Our participants often felt underrecognized for the patient’s workload and complexity of the care they performed. *(*[103]*– US Colorado)* | N=6  [40, 53, 59, 100, 103, 106] |
| **CMOC B8: When PAs are given sufficient time for skill development and to understand their clinical work environment (C), they feel comfortable and can develop their role (O) because they can gain the experience and knowledge needed to do their job (M).** | - The fact that she has been working on the ward for years, that she knows exactly how the ward functions, how everything works, what to do when there is a threat of overcapacity, what to do when tasks have been given to the nursing staff that are not executed in the right way, how to report incidents locally, she knows it all. She knows all the ins and outs of the ward, knows how everything works, so she is able to act quickly the moment she realises that something is not going well. *(*[32]*- Netherlands)* - Although the caseload was high, this out-of-hours work was described positively as offering opportunities to expand and develop the PA role: We realised when we first started working that the weekend and the evenings were the best time for us to get more experience . . . professionally it was really helpful because we could get more, we had more opportunities to practise our procedures because there were less trainees around . . . so, our lead consultant at the time . . . they wanted more cover on the weekends and some more cover out of hours, and then it kind of worked for us in terms of our own learning as well, so it’s kind of what we decided on. *(*[86]*– UK England)* - Across all settings, physicians and PAs provided examples of where being consistently present in their setting or working with particular patients allowed the PA to become a procedure or content expert due to frequency of exposure and clinical experience, or develop a skill set that extends physician services. *(*[91]*– Canada Ontario)* | N=16  [24, 32, 42, 59, 60, 65, 81, 86, 91, 93, 96, 100, 104, 106, 126, 131] |
| **CMOC B9: When clinical team members are given sufficient time to work with PAs and share physical spaces with PAs (C), they have more informed views about PA roles (O) because the exposure allows them to form clearer judgement about PA capabilities (M).** | - Many participants described positive changes over time in how their supervising doctor and medical/surgical team members perceived and accepted the PA role. In particular, participants reported that recognition their role offered continuity to staff and to patients resulted in them being used as a skills and knowledge resource. *(*[40]*– UK England)* - Over time, the experienced physician associates became viewed as a positive asset to medical and surgical teams, even in services where high levels of scepticism were initially expressed. *(*[56]*– UK England)* - As mentioned previously, PAs or NPs were formerly not part of the ID workforce. ID faculty and fellows have, however, interacted closely with them on the oncology services, as PAs and NPs are often the ones who initiate ID consultations and implement the resulting recommendations. These interactions built up a level of comfort and trust that enabled the ID department to incorporate PAs and NPs into its workforce. *(*[45]*– US Texas)* | N=14  [32, 35, 39, 40, 43, 45, 56, 65, 86, 91, 96, 99, 106, 133] |
| **CMOC B10: When PAs are given sufficient time to demonstrate their competency and reliability (C), supervisors trust them to carry out tasks as expected and reduce supervision (O) because they feel confident in their abilities (M).** | - The medical oversight of the PAs in this Pilot was considerable. The model of supervision was based on the supervision requirements of Australian junior medical staff and the practice of PA supervision in the USA. Medical supervisors decreased review requirements over time as PA competencies were demonstrated (from 100% of charts in the ﬁrst months to around 25%). The Pilot PAs, who were well qualiﬁed and very experienced, demonstrated a clear understanding of their own scope of practice and referred to the supervising doctor appropriately. *(*[65]*– Australia Queensland)* - Building trust in the competency of the individual PAs was a process reported by all three groups of doctors: “When our first PA started, clearly as doctors we weren’t entirely sure, I think a lot of us, what PAs could or could not do. And I think we’ve realised how competent our PAs are and how trustworthy, it’s been, you know, a revelation, we’ve been able to give them more and more jobs to do.” *(*[86]*– UK England)* - A larger number (91.9%) of supervising physicians agreed or strongly agreed that their PAs have been able to work more independently over time. *(*[99]*– Canada Ontario)* | N=10  [22, 56, 60, 65, 68, 85, 86, 91, 93, 99] |
| **CMOC B11:** **When PAs have previous professional background experience and understanding of the healthcare system (C), they are more likely to develop their role and identity (O) because they have confidence, understand workplace dynamics and can navigate team relationships (M).** | - This cohort of PA trainees with a paramedic background may also have benefited from an easier integration to the ED because of their familiarity with the environment from their previous work, especially working relationships with the nursing staff. *(*[42]*– Israel)* - The participants in this study reflected an increased attention to their own clinical competency in response to their exposure to workplace incivility. The improvement in clinical competency was associated with the self-perception of professionalism and a pathway to gaining respect. *(*[68]*– US Georgia)* - After the implementation of a structured onboarding program, there was an improvement in NP and PA awareness of health system expectation, policies, resources, and connectedness to peers. *(*[79]*– US California)* | N=6  [39, 42, 65, 66, 79, 113] |
| **CMOC B12:** **When PAs are given opportunities to develop their skills or engage in challenging work (C), they experience high satisfaction (O) because these opportunities align with their expectations and provide a sense of accomplishment, personal and professional growth (M)** | - Respondents rated most of their working conditions positively. The highest scores were accorded to the PA trainees’ sense that they were making significant contributions to the healthcare team and to the patients. They felt challenged and felt that they received high levels of responsibility. *(*[42]*– Israel)* - Physician assistant job satisfaction has remained relatively high in our academic cardiothoracic surgical program… Numerous variables of patient age (newborn, adult, or the elderly), diagnosis (congenital or acquired cardiac, pulmonary, or esophageal disease), and degrees of severity of illness (elective or emergent coronary or valvular heart disease, simple or complex congenital heart defects, benign or malignant pulmonary and esophageal diseases) allow the PA a wide array of work opportunities and skills. *(*[66]*– US Georgia)* - PAs felt that, if they took the initiative to learn more skills, that would be rewarded by increase of scope of practice and trust and appreciation from their team. *(*[116]*– UK England)* | N=17  [36, 40, 42, 51, 53, 66, 72, 74, 86, 91, 94, 100, 105, 113, 116, 119, 133] |
| **CMOC B13: When PAs are restricted in their scope of practice or assigned less desirable tasks (C), they experience dissatisfaction with their role (O) because they feel undervalued and unable to fully utilise their skills (M).** | - All participants impressed how not having the legal authority in England that they had in the US to prescribe medicines or order ionising radiation, had negative consequences for their role identity, with restrictions on the work they were given, and skills they were able to use. *(*[40]*– UK England)* - Failure to use the clinical knowledge and skills of CNPs, CNSs, and PAs affected overall morale and job satisfaction among many CNPs, CNSs, and PAs in our focus groups… “I feel like a cog in the bigger system sometimes where ‘See the patients, do your job, make us money, do as much, see as much, chart as much, bill as much as you can.’ And that gets to be a bit frustrating at times.” *(*[103]*– US Colorado)* - ‘The jobs’ Associated with the ward round in many cases, but ongoing throughout the day, were the activities that may be described as enabling patient care decisions – although these are described later in this study report as ‘clinical administration’ by others, no PAs used this term, but variously referred to these activities as ‘ward work’, ‘the jobs’ or ‘clinical jobs’. These activities did not involve direct patient contact, rather they took the form of ordering blood tests and investigational procedures – via a computer system, by telephone or occasionally in person; chasing the date for, or results of, such procedures; reviewing and acting on results; activities related to prescribing; and discharge summaries. In interviews, these ‘jobs’ were mentioned almost in passing by many, even though these were their core role, as if these did not require explanation or attention. *(*[86]*– UK England)* | N=12  [5, 40, 42, 46, 57, 86, 100, 103, 111, 113, 120, 121] |
| **BOUNDARY WORK** | | |
| **CMOC C1: When PAs are perceived as lacking experience, competence or understanding of the healthcare system (C), hospitals and departments may struggle to justify employing PAs (O) because they think they are less capable of meeting service needs (M)** | - “Any qualified PAs I’ve worked with have extremely limited medical knowledge, barely equivalent to a 2nd or 3rd year medical student at best…I’m not sure what function they are supposed to provide and certainly they should not be providing any unsupervised clinical care.” *(*[129]*– UK Nationwide)* - “They require a tremendous amount of training after PA school. Upon graduation, they can do little without assistance. Many physicians do not like new graduates because they create more work than they contribute for several months, if not years.” *(*[69]*- US - Virgin Island)* - The breadth and depth of medical knowledge, skill and judgement required for safe prescribing is significant and requires time to acquire. MAPs may not possess or be capable of this, especially PAs, as their training period is relatively short and their clinical experience limited *(*[122]*– UK Nationwide)* | N=24  [22, 25, 32, 39, 43, 53, 63, 65, 68, 69, 86, 91, 104, 106, 113, 122, 126, 129, 130, 135, 136, 139, 140, 142] |
| **CMOC C2: When PAs are introduced in settings where their roles are narrowly defined or restricted by regulation, governance or local protocols (C), hospitals, departments, and doctors may question their value (O) because they see the role as less effective and beneficial to service needs (M).** | - ‘‘I think we are incredibly hamstrung by the fact that the physician associates cannot prescribe or order investigations and without that their role is really quite limited and that is a huge, huge, huge impediment to going forward.” *(*[56]*– UK England)* - When exploring the barriers to recruiting PAs, the lack of regulation for PAs currently in Ireland had a clear influence. Due to this lack of regulation, PAs are not permitted to order ionising radiation or prescribe medications. Figure 3 shows that this situation was a deciding factor for the team or organisation. *(*[25]*- Ireland)* - “I feel strongly that PAs should be independent prescribers. Failure to (enable this) will limit the effectiveness of PAs within the NHS. I am aware that some NHS organisations are reticent to employ PAs due to their not being able to prescribe.” *(*[122]*– UK Nationwide)* | N=20  [22, 23, 25, 31, 40, 56, 57, 63, 65, 69, 86, 93, 96, 113, 120, 122, 130–132, 135] |
| **CMOC C3: When PAs work in settings with flexible or loosely defined regulations or policies (C), there is more scope for local adaptation of the role (O) because clinical teams are allowed to shape responsibilities around service needs (M).** | - The PAs’ capacity to adapt their role to address gaps within the local team was considered to be a benefit. In Mt Isa this was demonstrated through the establishment of a primary health care clinic to reduce non-urgent presentations to the ED.” *(*[65]*– Australia* *Queensland)* - Being able to develop a speciﬁc role or specialist contribution was frequently mentioned by participants as a signiﬁcant motivator to extending their contract. Several participants described a niche role of which they were proud for having taken on responsibility within their medical/surgical team. Examples included providing a pre-operative clinic for cardiac surgeons, and responsibility for an outpatient orthopaedic clinic to assist increased patient ﬂow. *(*[40]*– UK England)* - The GMC has argued that once registered, it simply requires AAs and PAs to work within their competence and that this requirement can be enforced without the need for any universal limit/restriction on what (outside legal restrictions such as prescribing) an AA or PA may do. The GMC has stated it can take a case-by-case approach to regulating the competence of AAs and PAs, and that it can assess whether a particular AA or PA has worked beyond their competence by having regard to guidance from employers, the Royal Colleges, and if necessary, an expert. *(*[131]*– UK Nationwide)* | N=51  [23, 24, 26–29, 31, 32, 35, 39–43, 45, 47, 49, 55, 59–61, 65, 66, 69, 72, 75, 80, 86, 91–93, 96, 98–103, 105, 108, 113, 117, 118, 120–122, 124, 126, 131, 134, 136] |
| **CMOC C4: When PAs work in settings where there are staffing challenges (e.g. rural areas, high patient demand, or emergencies) (C), by necessity they tend to have broader scopes of practices (O) because of a need to maintain service delivery (M).** | - As a pilot, hypothesis-generating study, our results indicate that EM PAs manage high-complexity conditions and perform complex procedures. PAs practicing in rural EDs appear to have an even greater scope of practice than urban PAs. We also found that rural PAs were more likely to work in EDs without onsite physician supervision, have less access to EM board-certiﬁed or any physician supervision if needed, and have fewer of their patients evaluated by physicians. *(*[70]*– US Nationwide)* - The trend toward expanded scope of practice for APPs is generally driven by issues with access to care. It is important to note the role of the COVID-19 pandemic in furthering the trend toward independent practice of APPs as most states temporarily expanded the scope of practice temporarily. *(*[120]*– US Nationwide*) - The strike cover for the gastroenterology ward this weekend will be PAs and a consultant. The PAs will be doing the role of a resident doctor. PAs in this department do not usually work weekends or out of hours. *(*[22]*– UK Nationwide)* | N=19  [22, 49, 50, 58, 64, 69–71, 75, 87, 92, 97, 99, 107, 116, 119, 120, 129, 136] |
| **CMOC C5: When flexibility and local variation in the professional scope of practice for PAs starts to introduce overlaps with other health care professional groups (C), there is confusion about the purpose of PAs in a service (O) because of ambiguous and unclear boundaries (M).** | - However, there is clearly some misunderstanding, mostly on the part of the junior residents, of how the NPPs ﬁt into the surgery team hierarchy. *(*[37]*– US Pennsylvania)* - An additional complex problem has been the issue of professional identiﬁcation. In our experience, the PAs tend to identify readily with the physician staff (i.e., residents and attendings) inasmuch as their responsibilities are most similar to those personnel. However, aspects of the professional situation, such as shift scheduling and institutional limitations on PA functioning, cause the role to resemble that of the nurses and other ancillary personnel. This has created some confusion for the PAs and for the administration concerning their role. *(*[59]*– US New York)* - In addition, the variability of the PA work led some staff, particularly in hospital 1, to ask for more clarity around the PA role within the team so that they can contribute to care more effectively. They [PAs] often end up doing a similar job to a junior doctor and that is perhaps not the best use of their training. I think departments needs to re- think how best PA's can be utilised to benefit the team and the PA's themselves. [Mid- grade doctor ID:3, hospital 1] *(*[53]*– UK England)* | N=28  [23, 37, 39, 40, 43, 47, 48, 51, 59, 69, 73, 86, 91, 99, 100, 103, 106, 116, 117, 129–131, 133–137, 140] |
| **CMOC C6: When the professional scope of practice starts to overlap with other established professional jurisdictions especially when other professionals are developing their roles (C), there is interprofessional resistance (O) because of concerns about professional encroachment (M).** | - A significant proportion of OR nurses (40%) felt that tasks performed by PAs in the OR fell within the scope of nursing practice…The nurses’ concern were not that the PAs were filling an expanded allied health care role that should be performed by nurses but, that during an operating room day, they were undertaking some activities that were traditionally performed by nurses. *(*[34]*- Canada Manitoba)* - The second example was from a clinic setting, in which a nurse practitioner reported that her work was changed by the presence of a PA. The following quotation reflects the ambiguity and overlap of roles there can be in patient care: When the PAs come in, the doctors automatically gravitate towards letting them see the patient over me. And maybe because as a nurse practitioner, and especially because I’m new and my role is new in this clinic, I think too that, you know like, I don’t sort of blame them because I think they would . . . they do have that much more knowledge base. However, they wouldn’t have a nursing input but then the nursing input could be substituted with any other nurse. *(*[86]*– UK England)* - Clinicians have expressed concern about the nature and degree of overlap of services provided by nonphysician clinicians and physicians. Are these two groups working in a complementary fashion, delivering the same services to different groups of patients; as collaborators, offering different services to the same patients; or as competitors, providing the same services to the same patients? *(*[89]*– US Nationwide)* | N=22  [5, 22, 23, 25, 30, 32, 34, 39, 42, 43, 50, 53, 56, 86, 89, 104, 120, 126, 127, 131, 132, 135] |
| **CMOC C7: When clinical team members have unrealistic or conflicting expectations for PAs (C), they are likely to be disappointed (O) because these expectations do not align with PAs’ actual capabilities (M).** | - Staff did not know how to relate to PAs. Nursing staff were unimpressed that PAs did not help with nursing duties or drug administration, and some doctors diverted the PAs from clinical tasks toward clerical duties. Many commented on their enthusiasm and valued an extra pair of hands, but said that they were of little clinical use. *(*[43]*– UK England)* - while enthusiasm should be encouraged, it is important that expectations for success are carefully managed. One of the challenges encountered in this project was that staff were not aware of the limitations of the new providers. As a result, there were some unrealistic expectations about their capabilities. *(*[73]*– Canada Ontario)* - One consultant argued that some PAs did not see themselves as assistants to the doctors, particularly those new to the team, but that the doctors did see them that way, and this caused tension. *(*[86]*– UK England)* | N=12  [23, 30, 42, 43, 59, 69, 73, 86, 102, 103, 111, 129] |
| **CMOC C8: When PAs’ roles and responsibilities require access to shared resources such as training opportunities, supervision, or funding (C), some clinical team members may feel that PAs compete for these scarce resources (M), leading to resistance to their integration (O).** | - 72.6% of doctors reported having "fewer" or "far fewer" training opportunities due to working with PAs. Core trainees and junior doctors reported significant displacement from practical learning experiences, with PAs frequently receiving supervised training over medical trainees. *(*[132]*– UK Nationwide)* - On the other hand, participants noted that PAs in the clinical learning environment may hinder trainee learning if PAs perform procedures that trainees need to learn or when PAs see patients with educational value for trainees. *(*[35]*– US Texas)* - The clinical teams (are) expected to train and supervise doctors in training, ACPs in training, and now PAs but without any extra supervision time with consultant PAs at max allowance for SPA of 0.5 and already go beyond this allocation supervising. PA teaching slots advertised by medical schools to clinicians without any pay for the hours to provide lectures and sessions. *(*[129]*– UK Nationwide*) | N=20  [22, 23, 32–35, 37, 43, 53, 86, 88, 104, 126, 129, 131, 132, 135–137, 140] |
| **CMOC C9:** **When PAs are perceived to have organisational advantage over other clinical team members, such as higher salary or better working hours (C), this creates dissatisfaction and resentment for other clinical team members (O) because they view it as unfair (M).** | - The basic pay for most PAs in the UK is more than that of a nurse or a doctor at the point of qualiﬁcation. This was identiﬁed as a point of contention which risked becoming a barrier to integration; PA (9): I’ve had quite a few times when people have brought up salary so I’ve tried to just avoid it. I don’t know, I ﬁnd it a bit awkward because it’s not down to us how much we get paid. Although this was an issue beyond the scope of individual organisations to directly address, participants reported examples of this being considered in ways that did not adversely impact integration. *(*[23]*– UK England)* - 96% of doctors believe it is unfair that newly qualified MAPs are paid 35% more than a first year (FY1) doctor for fewer hours per week and no on-call shifts (nights, 13 hour long days). *(*[129]*– UK Nationwide*) - For example, as most PAs worked core hours only during the week, this caused some friction amongst staff working shifts and weekends. *(*[53]*– UK England)* | N=13  [22, 23, 37, 42, 43, 53, 126, 127, 129–132, 135] |
| **CMOC C10: When PA roles does not compromise resources for other clinical team members (C), they are more accepting of PA roles (O) because they see PAs as an addition rather than displacing or undermining their own role (M).** | - The residents were nearly unanimous that PAs reduced their workload, and they generally felt that PAs relieved them of clinical responsibilities so that they could attend to teaching. Half of the residents agreed that “PAs facilitate their training experience during the arthroplasty rotation”; the other half were neutral but didn’t feel that PAs specifically improved their learning on the ward or in the OR. *(*[34]*- Canada Manitoba)* - Despite these and other initial nursing concerns regarding role deﬁnition, most nurses who worked with the PAs felt the health system could potentially beneﬁt from utilising PAs as additional mid-level clinicians, as long as opportunities for the nursing profession were not compromised. *(*[65]*- Australia – Queensland)* - Khakoo says that doctors working at his trust do not see the PAs as a threat. “They see them as a group of people who they work alongside and really help them.” PAs work particularly well alongside junior doctors, Khakoo says, because they are permanent members of staff who are able to build up the kind of organisational knowledge that junior doctors don’t have the time to acquire. *(*[134]*– UK Nationwide)* | N=16  [32, 34, 35, 37, 39, 41, 56, 59, 65, 66, 86, 114, 116, 126, 134, 135] |
| **CMOC C11: When breadth and flexibility in the scope of practice of PAs allow them to take on** **supportive or complementary tasks (e.g. extending clinical service functions or undertaking less desirable or low-priority clinical tasks) (C), they may gain recognition and acceptance from others (O) because they are perceived as beneficial to the team (M)** | - The addition of PAs to the CJRG team has allowed a single surgeon to run 2 rooms during a single operating day, increasing the volume from 3 to 7 primary joints per day. This was accomplished by using 1 physician assistant per room, with each room having its own dedicated nursing team and anesthesiologist. *(*[34]*- Canada Manitoba)* - They’re [PAs] just great at coming in and just taking off those little jobs that will really slow you down unnecessarily and paving the way for the more important sicker patients to get more of your time and attention. *(*[86]*– UK England)* - With the addition ofthe fourth PA in October 2008, the weekend service was initiated. Before this, a faculty and one fellow were in charge of all of the ID patients, including new consults on the weekend. Most patients could not be observed over the weekend. The PA weekend service allowed up to an additional 30 patient follow-ups to be performed... Over the past several months, the PA weekend service has helped ensure quality and continuity of care throughout the weekend. This has relieved the pressure on the faculty/fellow team considerably, allowing them to focus on new consultations and care of critically ill patients over the weekend. *(*[45]*– US Texas)* | N=14  [31, 32, 34, 37, 43, 45, 48, 53, 56, 69, 86, 89, 90, 136] |
| **ROLE PERCEPTION AND ACCEPTANCE** | | |
| **CMOC D1: When clinical team members have no or limited exposure to PA roles (C), this leads to confusion about PAs’ roles and responsibilities (M) which may result in resistance and reluctance to accept PAs as legitimate members of the team (O)** | - A hindering factor which was mentioned is that it regularly happens that physicians from other medical specialities demand to consult a physician instead of a PA about a patient. Related to this, the positioning of their profession was mentioned by PAs as an inﬂuencing determinant. *(*[32]*- Netherlands)* - Two main concerns were expressed by doctors and nurses regarding the role and its potential impact for the future. The ﬁrst was that PAs might impact negatively on medical training, either by reducing junior doctor learning opportunities or by increasing competition from a cohort of newly trained PAs who would also require medical supervision. These concerns diminished for doctors who worked with PAs and saw the role in practice. *(*[65]*– Australia Queensland)* - Across the board, those who are currently working with PAs in paediatric settings are significantly more likely to select that PAs’ delivery of these tasks is effective. This positivity is driven primarily by those with recent experience working with PAs in paediatric settings, who are statistically significantly more likely to agree that PAs can support teams in the delivery of high-quality care compared to those who do not recent experience. *(*[136]*– UK Nationwide)* | N=11  [22, 32, 34, 37, 65, 86, 91, 130, 136, 138, 141] |
| **CMOC D2** **When powerful stakeholders within a hospital who value the PA role are involved in setting PA role objectives and standard (C), they are more likely to facilitate PAs’ integration with the clinical team (O) because they have the authority to do so (M)** | - In addition, physicians who are involved in setting standards and designing objectives are more likely to embrace the change. Those sites that had physician leaders and physician involvement were more successful in the implementation of the new roles. *(*[73]*- Canada Ontario)* - Interview respondents acknowledged that Queensland Health had invested signiﬁcant time, care and resources into the Pilot, including consultation with medical and nursing peak bodies and colleges, full-day planning workshops, presentations to the staff at each site, the production of detailed documentation outlining the planned approach and roles, and the involvement of key medical ofﬁcers from each site in the recruitment and interview process. Other key factors included the leadership provided by medical supervisors and the extent of welcome and induction locally. The orientation process varied across sites but was crucial in determining the extent to which the PA was able to integrate into the healthcare team. ([65]– Australia Queensland) - Many participants identiﬁed the key role of dedicated supervisors in promoting integration, particularly in acting as an advocate, facilitating training and planning career development. *(*[23]*– UK England)* | N=9  [23, 29, 31, 51, 65, 73, 86, 109, 116] |
| **CMOC D3: When PA roles are institutionalised through policies, procedures, and established leadership structures (C), PA integration is more sustainable and coordinated (O) because these institutionalised structures create clear rights and obligations (M).** | - For those AMCs and hospital systems interested in replicating our process, there were several key takeaways that should be considered. First, create workgroups by clinical department comprised of APPs, physicians, practice managers, department administrators and other key stakeholders to review current state and consider strategies to optimize APP workflows and patient care goals. *(*[51]*– US California)* - A PA practice plan — detailing authority to prescribe medications, authority to order radiology and pathology tests, and level of supervision — was created and signed by the PAs and their supervisors in November 2008. *(*[62]*– Australia South Australia)* - Typically, detailed work on PAs, including governance arrangements, was taken on by designated PA boards or committees, which had sometimes had changes in leadership. MDs were commonly the link between the PA board and the trust board. *(*[86]*– UK England)* | N=25  [23, 27, 30, 33, 36, 37, 48, 51, 56, 59, 61, 62, 65, 69, 72, 77, 79, 86, 91, 100, 103, 106, 109, 122, 124] |
| **CMOC D4: When the PAs with previous professional background and experience can demonstrate relevant confidence and skills (C), they are viewed as a credible and legitimate addition to the clinical team (O) because others can appreciate their value (M).** | - The background and professionalism of the Pilot PAs were also undoubtedly important, as were the breadth and depth of their previous professional experience. PAs were speciﬁcally recruited for their experience and resilience in new situations. Consequently, participating PAs demonstrated clinical conﬁdence, skills and personality traits that enabled them to acclimatise quickly and to introduce a new role in an unfamiliar health system and culture. Newly qualiﬁed or less experienced PAs would not be likely to perform at an equal standard initially and would most likely require greater levels of supervision when ﬁrst employed. *(*[65]*– Australia Queensland)* - We recognize that the PA in our study was unique in having a background as a former comprehensive ophthalmologist. This prior experience, combined with a low, well-deﬁned threshold for calling for assistance, and ongoing supervision by our assistant chief of service and senior residents enabled the PA to serve as the primary consult respondent after just 6 weeks of direct supervision. *(*[41]*– US Maryland)* - A small number of respondents wanted the register to include information about previous qualifications of PAs and AAs, ordered by date of qualification. This included details of a PA or AA’s original degree used to gain entry to a PA or AA course, as well as any other qualifications obtained relating to the membership of another regulated health profession or registration with another regulator. *(*[126]*– UK Nationwide)* | N=13  [23, 32, 39, 41, 42, 59, 65, 68, 69, 86, 104, 120, 126] |
| **CMOC D5: When PAs are able to clearly, approachably and professionally communicate to others about their role (C), this leads to the PA being viewed as a credible and legitimate addition to the team (O), because others gain a better understanding of their role (M).** | - As well as linking the nursing and medical teams, as described previously, PAs were frequently described as an intermediary between the two. The reasons offered for this included PAs being more approachable and less intimidating: I quite like them in the sense that you always have this like nurse/doctor thing and they’re not really either if that makes sense so, it’s sort of, not a neutral party that’s not quite the right word, but they’re not a nurse or a doctor.… And it’s quite nice to have them there to ask, ‘cause you wouldn’t, like, bleep the SHO to be like ‘Can you just give me your opinion?’. But if they’re [the PAs] on the ward it’s quite nice to have them there, like every problem you had kind of went through her so that was, like, quite nice. *(*[86]*– UK England)* - Responses regarding types of workflow identified PAs as a vital communication bridge within the team based models … “We couldn’t care for patients without them [PAs]. [Our subspecialty] care is very complicated; it has a lot of moving parts. There’s more demand for services than we can accomplish just with physician and nursing staff. . . . essentially, they [PAs] have become the core of how subspecialty care is delivered in this institution. So operationally and logistically, it just isn’t feasible without PAs.” *(*[94]*– US Texas)* - PAs have a clear understanding of the PA role and can communicate that to others. PAs recognised quickly that it was up to them to be able to explain the scope of their role well to members of the healthcare team: Surg PA 28 – ‘I think the doctors, a lot of them didn’t know what to expect. I think I’ve shown them, [over time], what I am able to learn and take on…I’ve been very vocal about how I want to progress. I think the doctors are very much in support of that and so I think they’re happy to teach me and train me.’ *(*[116]*– UK England)* | N=13  [22, 30, 37, 58, 68, 69, 86, 91, 94, 113, 116, 129, 132] |
| **CMOC D6: When PAs’ relationships with the clinical team are strained (C), they may experience dissatisfaction with their role (O) because these experiences threaten their sense of belonging and professional identity (M)** | - Ernst and Kirsten both encountered physicians who refused to talk to them about a patient because they were PAs. They both described a feeling of anger and gave some explanation as to why they felt that particular emotion. “I was angry about it…it was hurtful…to have a colleague dismiss you …as if I have nothing to offer, even though I'm the one that just took care of your patient… so, I mean, it makes you angry, you're frustrated, but it also hurts…” *(*[68]*– US Georgia)* - The average job satisfaction score was 3.08 out of 5. Regarding subdomains of job satisfaction, the average work environment score was 2.58 out of 4. Regarding subdomains of job satisfaction, interaction had the highest score (3.86) and reward had the lowest score (2.39). *(*[111]*– South Korea)* - The APPs also found satisfaction in professional, social, and community interaction (0.72), including the quality of assistive personnel, social contact at work and after work, status in the community, professional interaction with other disciplines, interaction with other APPs, recognition of their work from peers, and acceptance and attitudes of physicians outside of the practice. *(*[36]*– US Florida)* | N=8  [30, 36, 68, 103, 105, 111, 119, 121] |
| **CMOC D7: When hospitals and departments foster a psychologically safe and supportive culture (C), PA integration become more effective and sustainable (O) because** **PAs and clinical team members are more willing to raise concerns and address operational challenges (M)** | - The study shows that a proactive approach by senior leaders and managers can help shape a culture where everyone feels valued and thus prevent development of negative attitudes. In order to further promote positive attitudes towards PAs and increase understanding of roles, opportunities for interprofessional learning throughout education and training can further improve how team members best work together to provide optimal healthcare for people as part of a system that is in much need for the kind of support PAs can offer. *(*[53]*– UK England)* - 26.3% of doctors strongly agree and 25.3% agree that “I practise defensively when engaging with associate roles because I believe I am working in a blame culture” *(*[130]*– UK Nationwide)* - The BMA’s February 2025 survey found that 75.2% of respondents were fearful of being unfairly blamed for errors involving associate roles in their workplace, with over half of all respondents practising defensively when engaging with associate roles because they believe they are working in a blame culture. ([131]*– UK Nationwide*) | N=7  [53, 86, 102, 120, 126, 130, 131] |
| **CMOC D8: When PAs become established and are perceived as reliable and authoritative within a team (C), this can inadvertently hinder the development of newer team members (O) because others may find it more productive to work with PA first (M)** | - However, trainee learning may be hindered if trainees are intimidated by experienced PAs on the team who know the system, patient population, clinical staff, and medical discipline better than do the trainees. As one PA (P09) said, this intimidation may interfere with trainees developing conﬁdence as physicians and may make it more difﬁcult for trainees to establish their own practice style instead of tending to conform to the PA’s way. *(*[35]*– US Texas)* - One potential danger to the teaching program is that attending physicians and nurses may get so dependent on the MLPs that the house staff may become “marginalized.” It is therefore important to have the MLP fully integrated with the team. In this model, the team rather than the individual attending physician manages the delegation. *(*[40]*– US Virginia)* - NP 860 – ‘Experienced’ or ‘longer term’ PA’s than rotating foundation doctors, who know the consultant team, going to theatre when time allows. No equal sharing of these opportunities with foundation doctors on the rotation. Pressuring and expecting rotating doctors to prescribe and request when told to, based off of PA assessment or judgement, and almost bullying if this is questioned. *(*[22]*– UK Nationwide)* | N=4  [22, 35, 39, 68] |
| **CMOC D9: When the PA role overlaps with traditional roles in healthcare (C), patients and the community struggle to differentiate between PAs and other clinicians (O) because the boundaries of these roles are not clearly defined or understood (M)** | - A mentioned determinant by the interviewees from the patient perspective was that many patients are not familiar with the PA professional in general, and that they often do not know whether they saw a physician or a PA. *(*[32]*- Netherlands)* - However, the introduction of the new PA role had also brought a lack of clarity, with patients not knowing if they were treated by a PA or a doctor. Also, it caused some confusion amongst existing roles. This became particularly evident as duties and training opportunities overlapped with other clinicians. *(*[53]*– UK England)* - Which of the following NHS professionals do you think are doctors? Specialty doctor 80%, junior doctor 79%, medical consultant, 68%, specialty registrar 48%, foundation doctor in training, 40%, associate specialist 31%, physician associate 25%, physician assistant 20%, don’t know 8% ([128]– UK Nationwide) | N=12  [32, 53, 54, 86, 95, 96, 104, 113, 122, 125, 127, 128] |
| **CMOC D10: When patients and the community have concerns about the PAs’ competency (C), they may not feel comfortable receiving care from PAs (O) because they lack confidence in the PAs’ ability to provide safe and effective care (M).** | - Seventy-nine per cent of the respondents stated that they would rather wait longer to be operated on by a doctor than being operated on earlier by someone who is not medically qualified but trained to perform the operation only. *(*[95]*– UK England)* - When asked, “If you had a choice of between being treated by a physician assistant or a physician for a major concern, which would you choose and why?” All 10 potential patient participants indicated they would prefer to be treated by a physician. The two main reasons cited for choice of a physician for a major concern included were competency (n = 3) and thoroughness (n = 4). *(*[69]*– US Virgin Island)* - Participants, particularly in the older groups, raised concerns over PAs/AAs’ level of autonomy and the critical, clinical nature of many tasks. Some were most concerned about AAs. This unease was rooted in a lack of trust in PAs’ and AAs’ abilities, and framed in the context of struggling healthcare systems. *(*[125]*– UK Nationwide)* | N=8  [53, 54, 56, 69, 95, 104, 113, 125] |
| **CMOC D11: When patients and the community do not fully understand the PAs’ role but trust the healthcare teams’ competence and care process (C), because they feel confident in the care they receive (M), they will be satisfied with the services provided (O)** | - Patients and relatives did not understand the PA role; however, they placed more emphasis on their own care on than the job title of individuals in the team treating them… All participants were happy to have a PA involved in their care in the future… Most described the PAs within a team context, which was delivering good care, and referring back to senior members of staff, and that was what was important rather than an individual’s title *(*[86]*– UK England)* - Regarding patient perceptions of satisfaction with APP care across ambulatory sites, we used results from surveys conducted by National Research Corporation (NCR health). For FY 22, our ambulatory APP cohort received a net promotor score (NPS) of 85.7 for recommend providers office, which shows that approximately 90% of patients are loyal and satisfied with the care they received and would continue to see that provider, refer others, and would speak highly of our organization. *(*[51]*– US California)* - The survey was administered to 25 patients, and responses were received from 24 (96%). Overall, the patients expressed very positive opinions of PAs (Table 6). Some patients specifically commented that PAs were helpful in providing information and explaining aspects of their care. *(*[34]*- Canada Manitoba)* | N=13  [24, 29, 34, 47, 51, 53, 56, 59, 86, 104, 122, 125, 128] |
| **CMOC D12: When patients and the community see PAs as offering advantages over doctors (e.g. quicker service or more time with patients) (C), they are more likely to consider PAs as a preferred option (O) because of relative advantage (M).** | - When asked whom they would prefer for treatment of a minor concern, two potential patients said physician, two said either physician or PA, and six said PA. The reasons given by the six potential patients for seeing a PA for a minor concern (a) less waiting time for a service (n = 3), (b) competency (n = 2), and (c) more time spent with the patient (n = 1). Three potential patient participants said they would prefer a physician because of a smaller chance of a physician missing a potentially major condition or ailment. *(*[69]*– US Virgin Island)* - Some participants described the PA as the ‘liaison’ and ‘a go-between’ the consultant and themselves. Comments were made about the PA helping the patients understand what the management plan was, why it might have changed and what it meant. Several patients commented on the PA’s helpful use of ‘layman’s’ language. *(*[86]*– UK England)* - All participant groups felt that patients have a better experience in hospital because the PAs spend more time with patients and listen to them… A key benefit highlighted was the consistent presence of PAs on the wards since this enabled them to get to know patients and vice versa. This was especially important when patients had frequent hospital visits as PAs got to know the patients and could therefore provide continuity of care. Patients really appreciate it when they see the same face every day, especially when the rest of the medical team could be changing. *(*[53]*– UK England)* | N=7  [53, 54, 69, 81, 86, 104, 113] |
| **EVIDENCE AND IMPACT** | | |
| **CMOC E1:** **When there is evidence that PAs save resources and improve efficiency (C), hospitals and departments are supportive of their role (O) because** **leaders and decision-makers perceive PAs can valuably contribute to meeting key operational goals (M)** | - The PA cardiology role was formally evaluated at the end of the one-year pilot…Income generation from the role from rapid access chest pain clinic (RACPC) referrals and earlier referrals to the cardiac catheter lab were key highlights that contributed to the efficiency of facilitated discharges *(*[31]*– UK England)* - In this study, PAs were found to “free up” 204 hours per year, or the equivalent of four 50-hour work weeks, for their supervising physician. This time could be used for other activities such as operating in a second theatre, other patient care–related duties, research activities or administrative work. Furthermore, surgical throughput was greatly enhanced: PAs allowed for use of the double room model, which increased the group’s surgical throughput of primary joint replacements by 42% over the preceding year and facilitated a reduction in median wait times from 44 weeks to 30 weeks. *(*[34]*– Canada Manitoba)* - Most of the managers indicated that having PAs as part of the medical team reduced the use of locum episodes.… From all of the managers’ perspectives, although the reduction in use of locum episodes reduced costs, more importantly it maintained efficiency and quality in the service and patient safety. *(*[86]*– UK England)* | N=12  [28, 31, 32, 34, 41, 44, 45, 56, 65, 69, 86, 104] |
| **CMOC E2: When hospitals or departments experience positive outcomes from PA roles (C), they are more likely to support and sustain the PA role (O) because** **these positive outcomes reinforce leaders and decision-makers’ confidence in the role’s value and impact (M).** | - Experiencing positive outcomes of PAs positively inﬂuence the sustainability of the implementation of PAs in inpatient care. Relative advantages that were mentioned were improved continuity, quality and effectiveness *(*[32]*- Netherlands)* - [the physician associate] understands the type of patients that we see and understands the management pathways that are required and is able to interdigitate with the medical staﬀ to facilitate care – enhancing of the quality of care across the service ... In the way we have experienced it, the person who’s been in post, has enhanced the ﬂuid running of the service as a whole. *(*[56]*– UK England)* - Eight MDs reported that their trust was expanding its number of PAs owing to a positive experience to date. *(*[86]*– UK England)* | N=26  [23, 24, 28, 31, 32, 34, 38, 39, 41, 44, 45, 56, 59, 62, 63, 65, 66, 73, 78, 86, 87, 94, 96, 116, 129, 134] |
| **CMOC E3: When PAs operate as part of a larger clinical team (C), their individual impact could be difficult to demonstrate (O) because** **service performance and patient outcomes are typically measured at the team level, not the individual level (M).** | - None of the participants was able to offer any completed internal quantifiable assessment of the impact of the PAs. Most considered that it was a very difficult task to single out one person or type of professional from what was a team activity: I think it would be extremely difficult to look at patient outcomes because you measure what teams do, you don’t measure what individuals do. *(*[86]*– UK England)* - Quantifying their billing alone is not an accurate measure as patients are often treated both by PAs and physicians who bill for these services…Although it did seem the providers increased productivity, it was hard to measure on the basis of billing alone. *(*[45]*– US Texas)* - There was limited available site-speciﬁc data on waiting times, length of stay, throughput and other indicators, which may have reﬂected some impact of the PA role on service delivery, as PA interactions were recorded under the supervisors’ provider number. *(*[65]*– Australia Queensland)* | N=13  [35, 39, 45, 49, 51, 56, 65, 73, 80, 86, 96, 102, 120] |
| **CMOC E4: When PAs contribute to service delivery such as team continuity and interprofessional collaboration (C), it is difficult to quantify their impact (O) because these outcomes** **are often described subjectively and qualitatively, and are hard to measure using traditional quantitative indicators (M).** | - The information about the perceptions of PAs was obtained through a qualitative process of questionnaire use and follow-up discussions. This method was chosen because it is very difficult to formally measure many of the aspects of PA practice that may be acknowledged as important, such as the impact on teaching, patient communication and potential overlap with nursing roles and duties. *(*[34]*– Canada Manitoba)* - None of the documents provided quantiﬁed details of the physician assistants’ impact on costs, such as locum doctors’ costs, or service eﬃciency. Neither were interviewees able to provide quantiﬁable evidence of the physician assistants’ impact on services although they could give examples of clinical audit activity undertaken by physician assistants leading to a service improvement change. *(*[56]*– UK England)* - Anecdotal reports suggested positive effects of the PAs’ presence on waiting times and other aspects of the patient and clinician experience, but there is no objective measurement to verify these. *(*[65]*– Australia Queensland)*Files\\528 - 0 references coded, 0.00% coverage | N=10  [34, 41, 51, 56, 59, 65, 83, 86, 94, 96] |
| **CMOC E5: When there is heterogeneity in the way PAs operate within clinical teams (C), it is difficult to evaluate their impact (O) because the variation in roles and models of care leads to inconsistent outcomes and challenges in measurement (M).** | - There was heterogeneity noted in how APPs operated within teams, including whether APPs had daily patient caps, evaluated patients and billed independently, and were incorporated into resident teams. Given this heterogeneity in how APPs are utilized, in combination with the limited literature on the subject, it is difficult to determine the best models for APP integration to achieve the highest quality patient care. *(*[55]*– US Nationwide)* - Variation in the utilization of PAs in the ED have also been found in previous studies in other countries, and it is common practice in places such as the USA for each hospital to determine its own PA scope of practice and to have its own credentialing agreements with its PAs, subject to adherence to governmental regulations. *(*[42]*– Israel)* - A variety of work settings were reported, most frequently inpatient wards, with work generally taking place during weekdays...PAs reported working within a variety of secondary care team staffing permutations, with the majority of these being interprofessional… Additionally, the variation in supervisory arrangements within the interprofessional setting for this new role is an issue that may warrant further attention. *(*[64]*– UK England)* | N=21  [24, 33, 35–37, 39, 42, 51, 53, 55, 57, 64, 65, 69, 80, 83, 86, 94, 102, 103, 106] |
| **CMOC E6: When different types of indicators** **(e.g. service delivery, efficiency, patient satisfaction, and health outcomes) are used to evaluate the impact of PAs (C), it is difficult to interpret their overall impact (O) because it is unclear whether these metrics are fit for purpose (M1) or how to combine them (M2)** | - Patients seen by PAs versus FY1s had a significantly longer LOS (52 min); 237 min vs 185 min, p<0.001 (95% CI 45.03 to 59.67). …PAs saw more patients in Majors and Resus while FY1s saw more UTC patients *(*[112]*– UK England)* - No significant difference between the two study arms was found on QALY and total costs. Explorative analyses showed a significant difference in costs for LOS in favour of the MD model, and significant differences regarding personnel costs in favour of the PA/MD model…. Our previous analysis showed increased provider continuity on the ward with the presence of a PA. This study shows that this increased continuity did not cause a decrease in costs, especially because of the higher costs for LOS. *(*[82]*- Netherlands)* - The outcome measurement reported as the most significant regarding evaluating clinical efficiency was the number of wRVUs generated, followed by the number of patients seen (n=4). wRVU generation is a quantitative way to calculate productivity and possible compensation for providers. Although each hospital may have different criteria for what counts as one wRVU, the function of the unit is universal. Interestingly, even though four of the five locations ranked wRVUs as the most significant way to measure clinical efficiency, several leaders alluded to the difficulty in using wRVUs as a measure of PA efficiency. *(*[94]*– US - Texas)* | N=11  [35, 37, 38, 80–82, 86, 94, 112, 115, 132] |
| **CMOC E7: When evaluations of PA roles are affected by inherent methodological challenges or highly context-specific factors (C), it is difficult to draw robust conclusion about PA impact (O) because findings are confounded or not generalisable (M)** | - Medical workforce research relies on surveys such as censuses or secondary data such as administrative files. Both have their advantages and limitations. Administrative data capture all workers employed but sacrifice candid responses that help shape attitudes, roles, and relationships. Research on public organizations reveals a substantial and growing body of empirical evidence relevant to many international issues in political economy and organization theory, such as the privatization of public services. However, certain assumptions are made that may mislead goals. While the institutional data we obtained have a great deal of integrity due to accurate compensation and benefit structure, the data do not capture the role delineation of PAs employed in federal service. *(*[61]*– US Nationwide)* - The non-randomized character of this study implies an increased risk for confounding, which we took into account in the multivariable analyses. However, we cannot exclude that local differences like policies about quality of care and patient case-mix could have influenced our results. *(*[81]*- Netherlands)* - The limited evidence regarding the ability of PAs to practise within the NHS is based on small-scale quality improvement projects or local service evaluations. From our perspective, these do not provide the robust evidence needed to fully evaluate the safety and effectiveness of PA roles. It is also worth noting that it is unlikely that evaluations, led by departments who have invested in PAs, showing worsening metrics would be submitted for public appraisal in abstract or manuscript form: there is a particularly high risk of publication bias. More rigorous, unbiased research without conflicts of interest is needed to establish the effectiveness and safety of PA practice beyond the scope as set in the RCP interim scope guidance for PAs working in the medical specialities. ([135]– UK Nationwide) | N=8  [54, 61, 65, 80, 81, 110, 132, 135] |
| **CMOC E8: When organisations lack appropriate systems to monitor PA activity and outcomes (C), they are unable to provide robust and transparent evidence about PA role (O) because it is difficult to track and attribute PA’s specific contribution (M)** | - What renders this more challenging is that AMCs have not been systematically gathering data on outcomes, including mortality, ICU transfers, readmissions, relative value units generated, estimated associated costs, ratings of physician burnout, and patient satisfaction, for teams staffed by APPs compared with traditional team models. *(*[55]*– US Nationwide)* - None of the managers or clinicians, in any of the sites, was able to provide any routine data or reports from which the impact of the involvement of PAs could be considered. This absence of data has also been reported from surveyed executives of medical organisations in the USA, who noted the contrast to their primary care services, and similarly could not provide information on patient outcomes or costs that could be attributed to the inclusion or exclusion of PAs in hospital services. *(*[86]*– UK England)* - Further, not all institutions were able to provide details on the number of employed PAs at their institution which suggests that hospitals are not actively tracking this data. *(*[94]*– US Texas)* | N=7  [47, 55, 61, 86, 94, 108, 121] |
